# *Cinnamomum* Species: Bridging Phytochemistry Knowledge, Pharmacological Properties and Toxicological Safety for Health Benefits

**DOI:** 10.3389/fphar.2021.600139

**Published:** 2021-05-11

**Authors:** Javad Sharifi-Rad, Abhijit Dey, Niranjan Koirala, Shabnum Shaheen, Nasreddine El Omari, Bahare Salehi, Tamar Goloshvili, Nathália Cristina Cirone Silva, Abdelhakim Bouyahya, Sara Vitalini, Elena M. Varoni, Miquel Martorell, Anna Abdolshahi, Anca Oana Docea, Marcello Iriti, Daniela Calina, Francisco Les, Víctor López, Constantin Caruntu

**Affiliations:** ^1^ Phytochemistry Research Center, Shahid Beheshti University of Medical Sciences, Tehran, Iran; ^2^ Facultad de Medicina, Universidad del Azuay, Cuenca, Ecuador; ^3^ Department of Life Sciences, Presidency University, Kolkata, India; ^4^ Department of Natural Products Drugs Discovery, Dr. Koirala Research Institute for Biotechnology and Biodiversity, Kathmandu, Nepal; ^5^ Department of Botany, Lahore College for Women University, Lahore, Pakistan; ^6^ Laboratory of Histology, Embryology and Cytogenetic, Faculty of Medicine and Pharmacy, Mohammed V University in Rabat, Rabat, Morocco; ^7^ Medical Ethics and Law Research Center, Shahid Beheshti University of Medical Sciences, Tehran, Iran; ^8^ Institute of Botany, Plant Physiology and Genetic Resources, Ilia State University, Tbilisi, Georgia; ^9^ Department of Food Science, Faculty of Food Engineering (FEA), University of Campinas (UNICAMP), Campinas, Brazil; ^10^ Laboratory of Human Pathology Biology, Faculty of Sciences, Genomic Center of Human Pathology, Faculty of Medicine and Pharmacy, Mohammed V University of Rabat, Rabat, Morocco; ^11^ Department of Agricultural and Environmental Sciences, Milan State University, Milan, Italy; ^12^ Department of Biomedical, Surgical and Dental Sciences, Milan State University, Milan, Italy; ^13^ Department of Nutrition and Dietetics, Faculty of Pharmacy, University of Concepcion, Concepcion, Chile; ^14^ Universidad de Concepción, Unidad de Desarrollo Tecnológico, UDT, Concepcion, Chile; ^15^ Food Safety Research Center (salt), Semnan University of Medical Sciences, Semnan, Iran; ^16^ Department of Toxicology, University of Medicine and Pharmacy of Craiova, Craiova, Romania; ^17^ Department of Clinical Pharmacy, University of Medicine and Pharmacy of Craiova, Craiova, Romania; ^18^ Department of Pharmacy, Faculty of Health Sciences, Universidad San Jorge, Zaragoza, Spain; ^19^ Instituto Agroalimentario de Aragón-IA2 (CITA-Universidad de Zaragoza), Zaragoza, Spain; ^20^ Department of Physiology, "Carol Davila" University of Medicine and Pharmacy, Bucharest, Romania; ^21^ Department of Dermatology, "Prof. N.C. Paulescu" National Institute of Diabetes, Nutrition and Metabolic Diseases, Bucharest, Romania

**Keywords:** Ciannamomum spp., phytochemistry, Pharmacology, mechanisms of action, clinical trials, Toxicological data

## Abstract

The genus *Cinnamomum* includes a number of plant species largely used as food, food additives and spices for a long time. Different traditional healing systems have used these plants as herbal remedies to cure diverse ailments. The aim of this comprehensive and updated review is to summarize the biodiversity of the genus *Cinnamomum*, its bioactive compounds, the mechanisms that underlie the pharmacological activities and molecular targets and toxicological safety. All the data in this review have been collected from databases and recent scientific literature including Web of Science, PubMed, ScienceDirect etc. The results showed that the bioactive compounds of *Cinnamomum* species possess antimicrobial, antidiabetic, antioxidant, anti-inflammatory, anticancer and neuroprotective effects. The preclinical (*in vitro*/*in vivo*) studies provided the possible molecular mechanisms of these action. As a novelty, recent clinical studies and toxicological data described in this paper support and confirm the pharmacological importance of the genus *Cinnamomum.* In conclusion, the obtained results from preclinical studies and clinical trials, as well as reduced side effects provide insights into future research of new drugs based on extracts and bioactive compounds from *Cinnamomum* plants.

## Introduction

The *Cinnamomum* plants have been studied for its phyto-constituents and pharmacological properties as well as traditional medicinal significance. *Cinnamomum verum*, known as the “true cinnamon tree” and “Ceylon cinnamon tree” is an evergreen small, tree that belongs to the Lauraceae family. Along with other cinnamon species, such as *Cinnamomum cassia*, *Cinnamomum verum* etc., the tree bark is used to obtain cinnamon (Ribeiro-Santos et al., 2017). The ancient botanical name of this tree – *Cinnamomum zeylanicum*-derives from Ceylon the old name for Sri Lanka (Ribeiro-Santos et al., 2017). *Cinnamomum cassia*, also called “Chinese cinnamon,” is an evergreen tree, native to South China; Chinese cinnamon being produced mainly in the southern regions and is also widely grown in the other areas of the South and East Asia (Bedigian, 2005). People used cinnamon obtained from a variety of *Cinnamomum* plants as a spice from ancient times. It is mentioned in the texts written in Sanskrit and in the Bible, as well as in the works of Herodotus and Pliny (Lu et al., 2011). In Egypt, *Cinnamomum zeylanicum* was used in the embalming process. It was also added to foods for preservation. Both in India and Europe, *Cinnamomum* species have been traditionally used in the treatment of respiratory viruses, especially combined with ginger (*Zingiber officinale*). Ginger stimulates blood circulation in the extremities (toes, fingers), and *Cinnamomum* plants are an alternative natural medicine for reducing muscle pain and other signs and symptoms of colds and flu. Other traditional uses include urinary tract infections, relieve the abdominal discomfort, and improves digestion, antidiabetic, analgesic, and neuroprotective effects (Ravindran et al., 2003).

All types of cinnamon contain the active ingredient cinnamaldehyde, which accounts for between 65 and 80% of the essential natural oil. Cinnamon is used in case of dyspepsia, flatulence, nausea, intestinal colic, slow digestion, diarrhea, and digestive atony. This antispastic effect is attributed to the natural chemical compound catechin that contributes to the reduction of nausea and vomiting. Also, its volatile oil can help better food processing by breaking down fat during digestion. The studies showed that cinnamon helps diabetic patients to metabolize sugar more easily. In the case of people with type II diabetes, the pancreas produces insulin, but their body cannot use it effectively for decreasing blood sugar concentration (Chen et al., 2012). The researchers have found in recent studies that cinnamon improves insulin’s ability to metabolize glucose, helping to control blood sugar levels. It contains the antioxidant glutathione and a type of flavonoid called methyl hydroxychalcone polymer (MHCP) (Qin et al., 2010). The potential antidiabetic mechanism of cinnamon is associated with increasing the receptivity of adipose cells to the hormone insulin that regulates the metabolism of glucose and controls the level of sugar in the blood.

Cinnamon helps reduce pain due to its action of inhibiting prostaglandin. Cinnamaldehyde acts against the coagulation of platelets in the blood, which can hinder blood flow. It inhibits arachidonic acid’s release (a trigger for the inflammatory response) from cell membranes (Vallianou et al., 2019). Therefore, cinnamon is useful for the pharmacotherapy of inflammatory diseases, such as rheumatoid arthritis (Rogoveanu et al., 2018; Shishehbor et al., 2018). Recent studies showed that the scent and aroma of cinnamon act as cognitive stimuli, which could improve memory, visual-motor capacity and virtual memory (Nussbaum et al., 2017), due to its compound cinnamic aldehyde. Experimental researches on rodents receiving cinnamic aldehyde have reported improvements in stress-induced depressive behaviors. Cinnamic aldehyde is administered orally in the treatment of behavioral and mental disorders. Recent findings in this regard may also be helpful in treating depression (Panickar et al., 2009).

Starting from the ethnopharmacological premises of the therapeutic beneficial effects of Cinnamomum genus, in this manuscript were highlighted, based on scientific evidence the potential current pharmacological mechanisms and human clinical studies for the benefits of human health. Therefore, this current work reviews the comprehensive and current knowledge on the phyto-constituents, potential mechanisms of the main pharmacological activities evidenced by preclinical studies (*in vivo, in vitro*), recent clinical trials and toxicological data regarding safety of *Cinnamomum* plants.

## Review Methodology

Scientific search engines Medline, PubMed, ScienceDirect and Scopus were searched to retrieve literature and cross-references using key words: “*Cinnamomum*,” “phytochemistry,” “pharmacology”; “mechanisms of action”; “toxicology.” We included literature in relation to the bioactive compounds, pharmacological activities and underlying mechanism of action, clinical studies, toxicological and safety considerations of different *Cinnamomum* species. Plants’ taxonomy was validated using The Plant List (http://www.theplantlist.org/), and chemical formulas were validated by consulting the PubChem chemical base data (http://pubchem.ncbi.nlm.nih.gov/search/#collection=compounds). Inclusion criteria: *in vitro*/*in vivo* pharmacological studies using cell lines and laboratory animals, studies involving extracts of the genus Cinnamomum, studies with obvious mechanisms of action. Exclusion criteria: studies that included homeopathic preparations and other associated nutritional supplements, studies without explaining the mechanism of action.

## Phytochemistry of *Cinnamomum* Genus

### Chemical Composition

The chemical composition of cinnamon EOs (essential oils) varies depending on several factors that include the part of the plant used, growing season, age of trees, location, and extraction methods (Kaul et al., 2003; Barceloux, 2009; Wang et al., 2009; Chakraborty et al., 2015). Cinnamaldehyde and its analogs, butanolides, diterpenoids, lignans and several other compounds, are present in this genus. From the genus *Cinnamomum,* a total of 127 chemical compounds have been identified (Zhao and Ma, 2016).

**FIGURE 1:**
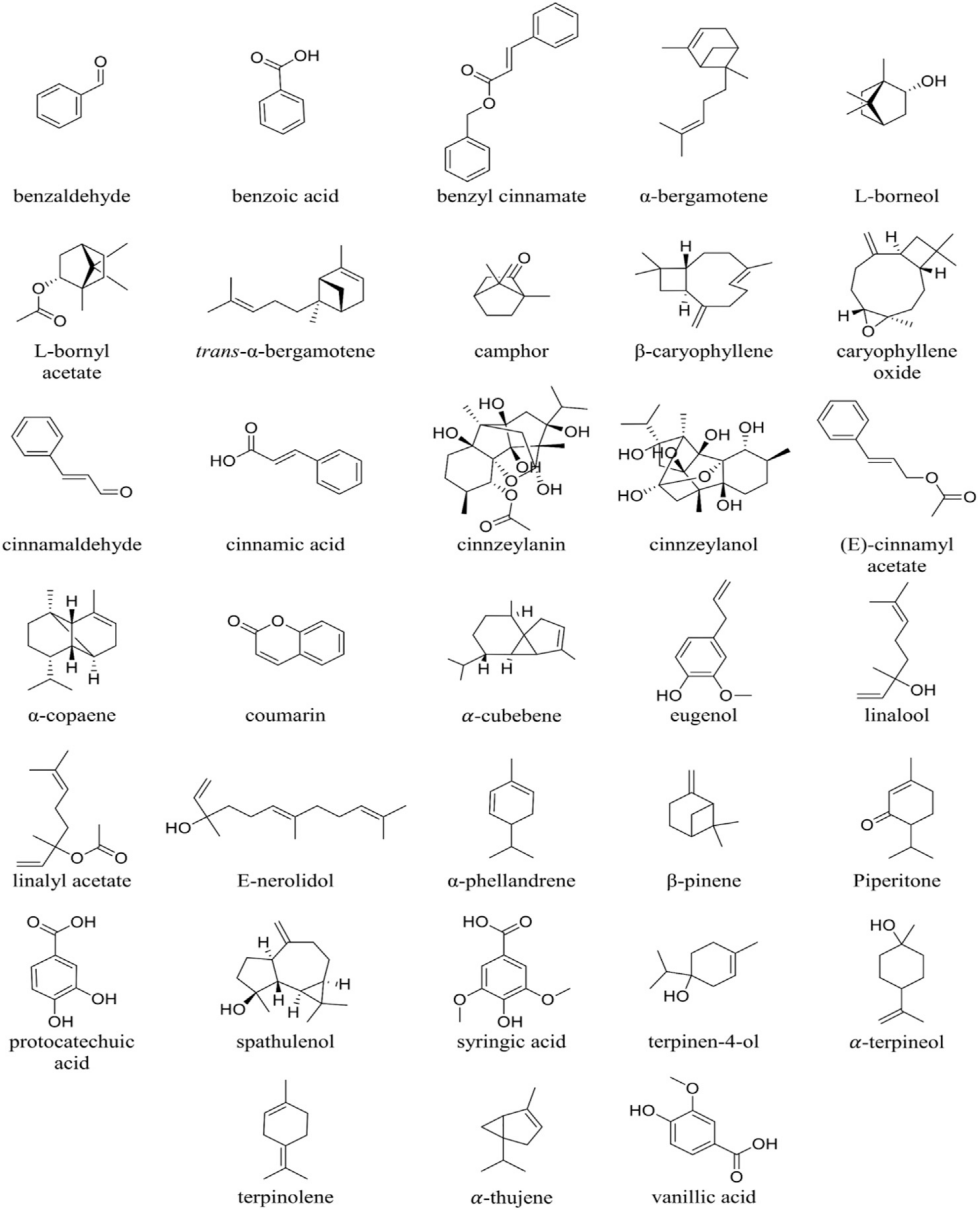
Chemical structures of **t**he phyto-constituents of *Cinnamomum* species.

Cinnamon presents a diversity of resinous compounds, including cinnamaldehyde, cinnamates, cinnamic acid and natural EOs (Senanayake et al., 1978) (Figure 1; Table 1). Over time, cinnamaldehyde changes color, absorbs oxygen and thus explains the appearance of the perfume and spicy taste (Singh et al., 2007). EOs contain a great variety of volatile natural compounds, such as *trans*-cinnamaldehyde, eugenol, cinnamyl acetate, L-borneol, *ß*-caryophyllene, caryophyllene oxide, L-bornyl acetate, *a*-thujene, *a*-terpineol, *a*-cubebene, terpinolene and E-nerolidol (Jantan and Goh, 1992; Jantan et al., 2003; Jantan et al., 2005; Chua et al., 2008; Abdelwahab et al., 2010; Tung et al., 2010; Geng et al., 2011). Spathulenol was reported as the major compound in leaf oil of *Cinnamomum altissimum* Kosterm (Jantan et al., 2003).

**TABLE 1:** The most representative chemical compounds of *Cinnamomum* plants.

Plant parts	Compounds	Ref
Bark	cinnamaldehyde 65–80% eugenol 5–10%	Yanakiev. (2020)
Bark of root	camphor 58%	Rao and Gan. (2014)
Leaf	eugenol 70–90%, cinnamaldehyde 1–8%	Utchariyakiat et al. (2016)
Fruits	*trans*-cinnamyl acetate 40–50% caryophyllene 10–15%	Bakar et al. (2020)
Buds	α-bergamotene 27% terpene hydrocarbons 80% α-copaene 20%, terpenoids 10%	Barceloux. (2009)
Flowers	*trans*-cinnamyl acetate 40% *trans*-α-bergamotene 10% caryophylleneoxide 8%	Bakar et al. (2020)

According to (Wang et al., 2009), eugenol (80%) is the main volatile compound in the EO of *Cinnamomum verum* J. Presl (synonym: *Cinnamomum zeylanicum* Blume) instead of *trans*-cinnamaldehyde (16.25%) and the other constituents such as: alcohols, aldehydes, ketones, alkanes, sulfides, and ethers. The chemical composition in the bark and leaf EOs of *C. verum* consists of high levels of eugenol (90.2%) and cinnamaldehyde (44.2%). The chemical constituents of *C. verum* bark EO include three major compounds and six minor chemical derivatives (Yang et al., 2012). Cinnamaldehyde (59%), benzaldehyde (12%) and eugenol (5%) are the major compounds, while the six minor constituents are α-phellandrene (1.1%), linalool (1.1%), benzoic acid (0.8%), β-caryophyllene (0.7%), linalyl acetate (0.6%) and benzyl cinnamate (0.6%). The most important compounds identified in the leaf oil of *C. verum* grown are eugenol (75%), linalool (8%) and piperitone (2.5%) (Raina et al., 2001).

Chemical constituents in the leaf EO of *Cinnamomum burmanni* (Nees & T. Nees) Blume are *trans*-cinnamaldehyde (60%), eugenol (18%) and coumarin (14%). Other constituents identified in the oils are alcohols, aldehydes, and ketones. The major components in the stem bark oil of *Cinnamomum iners* (Reinw. ex Nees & T. Nees) Blume are 1,8-cineole (41%), α-terpineol (15%) and terpinen-4-ol (14%). The other components identified are β-pinene (4.75%), γ-terpinolene (1.61%) and caryophyllene oxide (4.37%) (Wang et al., 2009).

Other minor constituents reported in cinnamon EO include: cinnamic acid, phenolic acids, oligopolymeric procyanidins, pentacyclic diterpenes, cinnzeylanol, and its acetyl derivative cinnzeylanine, mannitol, xylose, arabinose, xylanose, glucose, mucilage polysaccharides (European Scientific Cooperative on Phytotherapy, 2003). Several nonvolatile compounds have been found in cinnamon EOs such as cinncassiols, cinnzeylanol, cinnzeylanin, anhydrocinnzeylanol, anhydrocinnzeylanin, several benzyl isoquinoline alkaloids, cinnamic acid, β-sitosterol, flavanol glucosides, coumarin, protocatechuic acid, vanillic acid and syringic acid (Leela, 2008).

### Cinnamomum’s Natural Compounds: Pharmacokinetics, Bioavailability, Bioactivity and Metabolism

Considering the pharmacokinetics, bioavailability and metabolism of Cinnamomum active ingredients, very little works have been performed. Many studies described the role of bioactive compounds from the plants in enhancing bioavailability of some standard drugs (Salehi et al., 2020b).

Pharmacokinetics of cinnamic acid (CA) indicated the CA was readily absorbed and then metabolized quickly into hippuric acid (HA) when a decoction of *Ramulus Cinnamomi* (RC) [containing CA 7.62 × 10^–5^ mol/kg and cinnamaldehyde (CNMA) 1.77 × 10^–5^ mol/kg] was administered in rats via oral route. CNMA was found to be metabolized partially in stomach and small intestine into CA and almost fully metabolized in liver into CA before being absorbed into rat blood. The results indicated that plasma CA in RC group probably came from CNMA transformation in RC (Chen et al., 2009). Cinnamon also increased pioglitazone bioavailability via CYP3A4 enzyme inhibition in rat following oral administration owing to its possible use in combination with pioglitazone against diabetes (Mamindla et al., 2017). CA significantly inhibited rosuvastatin (RSV) (a specific breast cancer resistance protein) transport into rat bile thus enhanced the plasma exposure of the same (Basu et al., 2013).

In a recent study, comparative pharmacokinetic analysis of *Cinnamomum cassia* twigs’ standard decoction and dispensing granules containing three phenolics such as cinnamic acid, vanillic acid and protocatechuic acid in rats revealed the AUC_0–*t*_ values of the compounds by LC–MS/MS (Tao et al., 2019).

## Preclinical Studies Related to Pharmacological Activities and Potential Mechanisms of *Cinnamomum’*s Phyto-Constituents

Various traditional uses of *Cinnamomum* plants motivated a series of experimental investigations of the plant’s pharmacological properties (Wang et al., 2020). Those experimental approaches tempted to validate the potential uses of these plants as therapeutic remedies. Studies have been reported on extracts and isolated compounds of *Cinnamomum* plants, investigating antibacterial, anti-diabetes, anti-inflammatory, antioxidant, antitumor, and neuroprotective properties.

### Antibacterial Activity

In order to inhibit the growth and proliferation of pathogenic microorganisms, synthetic drugs have been commonly used for the treatment of microbial infections. The overuse of conventional antibacterial drugs could lead to serious side effects, including the selection of resistant bacterial strains and the development of antibiotic resistance during treatment, posing a real threat to global public health (Russell, 2002; Goyal et al., 2008; Călina et al., 2017; Ungureanu et al., 2017; Zlatian et al., 2018). Therefore, it is necessary to discover new sources of antibiotics such us natural antimicrobial compounds that could be an effective and cheaper alternative (Mbwambo et al., 2007; Salehi et al., 2019a).

**FIGURE 2:**
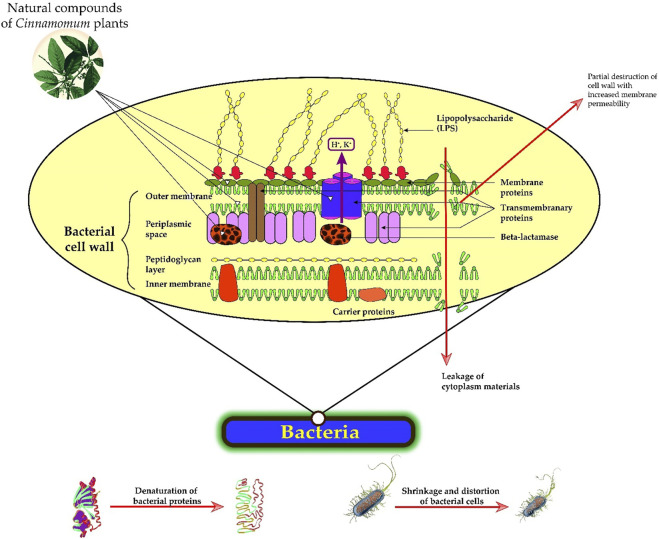
Antibacterial properties of *Cinnamomum* plants’ derivatives. The main potential mechanisms of antibacterial action are related to: 1) the partial degradation of the bacterial cell wall, 2) the increase of membrane permeability, 3) the leakage of cytoplasm materials, 4) the shrinkage of bacterial cells and prominent distortion, and 5) the alteration of secondary, tertiary structures and bacterial protein.

The mechanisms underlying the antibacterial effects of natural derivatives of *Cinnamomum* plants are complex. Cinnamaldehyde, as well as eugenol, inhibited β-lactamase’s production by the bacterium and destroyed its cell wall (Helander et al., 1998; Di Pasqua et al., 2007). Phenolic compounds such as carvacrol can also cause destruction of the cell cytoplasmic membrane (Ultee et al., 1999), and terpenes interact with the bacterial membrane by modifying its permeability (Lambert et al., 2001) and increasing the penetration of antibacterial agents. Essential oils of *Cinnamomum* contain a wide range of different groups of chemical compounds, suggesting that their antibacterial activity might have several mechanisms (Skandamis and Nychas, 2001; Carson et al., 2002). (Figure 2).

A recent study highlighted that MBC (minimum bactericidal concentration) and MIC (minimum inhibitory concentration) of the methanol extract of *C. burmanni* leaves were 625 and 2,500 μg/ml, against *Bacillus cereus*. For other bacterial strains (*Staphilococcus aureus*, *Listeria monocytogenes, Escherichia coli,* and *Salmonella anatum*), the values are >2,500 *μ*g/ml for both concentrations (Shan et al., 2007).

The methanol extract of *Cinnamomum tamala* (Buch.-Ham.) T. Nees & Eberm. stem bark revealed MIC values of 256, 4,096, 4,096, and 2048 *μ*g/ml against *Staphilococcus aureus*, *Streptococcus pyogenes*, *B. cereus* and *Bacillus subtilis*, respectively, with inhibition diameters ranging from 14.0 to 20.8 mm (Goyal et al., 2009). The aqueous extract of *C. verum* bark demonstrated antibacterial activity against *Moraxella cattarhalis* with MIC and MBC values of 120 and 240 mg/ml, respectively, and an inhibition zone of 11 mm (Rasheed and Thajuddin, 2011). (Table 2).

**TABLE 2:** Preclinical pharmacological activities of *Cinnamomum* genus.

Pharmacological activity	Cinnamum plant/extracts/fractions	Methods	Models cellular lines (*in vitro*)/animal (*in vivo*)	Effects/underlying mechanisms	Ref
**Antimicrobial**	*Cinnamomum altissimum/*stem bark/EOs	Disk diffusion	MRSA	IC_50_ = 12.0 mm	Buru et al. (2014)
				↓bacterial growth	
		Microdilution	MRSA	IC_50_ = 156.25 μg/ml	
				↓ bacterial growth	
	*Cinnamom umbejolghota*/leaves/EOs	Disk diffusion	*Escherichia coli*	19.5 mm	Wannissorn et al. (2005)
			*Salmonella saintpaul*	17.5 mm	
			*Salmonella derby*	16.5 mm	
			*Salmonella gallinarum*	34 mm	
			*Salmonella schwarzergrund*	16.5 mm	
			*Salmonella mbandaka*	18 mm	
			*Salmonella monterideo*	16.5 mm	
	*Cinnamom umburmanni* Leaves/EOs	Microdilution	*Staphylococcus aureus*	IC_50_ > 2,500 μg/ml	Shan et al. (2007)
			*Listeria monocytogenes*	IC_50_ > 2,500 μg/ml	
			*Salmonella anatum*	IC_50_ > 2,500 μg/ml	
			*Escherichia coli*	IC_50_ > 2,500 μg/ml	
	*Cinnamomum cassia* leaves/EOs	Microdilution	*Pseudomonas putida*	IC_50_ = 500 mg/L	Oussalah et al. (2006)
	*Cinnamomum cassia* leaves/EOs	Microdilution	*Bacillus cereus*	IC_50_ = 500 mg/L	Turgis et al. (2012)
			*Escherichia coli*	IC_50_ = 500 mg/L not determined	
			*Staphylococcus aureus*		
	*Cinnamomum cassia*/shoots/methanol	Disk diffusion	*Escherichia coli*	13 mm	Kim et al. (2004)
		Microdilution		IC_50_ = 250–500 μg/ml	
	*Cinnamomum cassia* bark/EOs	Disk diffusion	*Listeria monocytogenes*	22.4 mm	de Oliveira et al. (2012)
		Microdilution		IC_50_ = 0.03 μg/ml	
		Agar disc diffusion	*Bacillus subtilis*	21.1 mm	(Huang et al., 2014)
			*Salmonella typhimurium*	14.5 mm	
			*Staphylococcus aureus*	27.5 mm	
		Microdilution	*Staphylococcus aureus*	IC_50_ = 2.5–5 mg/ml	
		Microdilution assay	*Bacillus subtilis*	IC_50_ = 10 mg/ml	
			*Salmonella typhimurium*	IC_50_ = 20 mg/ml	
			*Escherichia coli*	IC_50_ = 10 mg/ml	
		Permeability of cell membrane	*Staphylococcus aureus*	↑ permeability of wall cell	
			*Escherichia coli*		
	*Cinnamomum chemungianum* leaves/EOs	Disk diffusion	*Staphylococcus aureus*	7 mm	Rameshkumar et al. (2007)
			*Bacillus subtilis*	8 mm	
			*Salmonella typhi*	9 mm	
			*Escherichia coli*	12 mm	
			*Pseudomonas fluorescens*	7 mm	
			*Proteus vulgaris*	7 mm	
			*Klebsiella pneumoniae*	11 mm	
	*Cinnamomumim pressicostatum* stem bark/VO	Disk diffusion	MRSA	14.5 mm	Buru et al. (2014)
		Microdilution		IC_50_ = 156.3 μg/ml	
	*Cinnamomum iners* stem bark/VO	Disk diffusion	MRSA	10.5 mm	Buru et al. (2014)
		Microdilution		IC_50_ = 625.0 μg/ml	
	*Cinnamomum longepaniculatum* leaves/VO	Microdilution	*Escherichia coli*	IC_50_ = 3.1 μL/ml	Li et al. (2014)
			*Salmonella enteritidis*	IC_50_ = 6.3 μL/ml	
			*Staphylococcus aureus*	IC_50_ = 6.3 μL/ml	
	*Cinnamomummicranthum* leaves/EOs	Diffusion method	*Vibrio parahemolyticus*	2 mm	Yeh et al. (2009)
			*Vibrio alginolyticus*	3 mm	
			*Vibrio alginolyticus*	3 mm	
			*Vibrio vulnificus*	2 mm	
			*Lactococcus garvieae*	1 mm	
			*Debaryomyces hansenii*	1 mm	
			*Photobacteria damsel*	1 mm	
			*Streptococcus* sp	1 mm	
			*Eromonas hydrophila*	2 mm	
	*Cinnamomum micranthum* twig/EOs	Diffusion method	*Vibrio parahemolyticus*	5 mm	
			*Vibrio alginolyticus*	5 mm	
			*Vibrio alginolyticus*	6 mm	
			*Vibrio vulnificus*	5 mm	
			*Lactococcusgarvieae*	3 mm	
			*Debaryomyces hansenii*	4 mm	
			*Photobacteria damsel*	7 mm	
			*Streptococcus* sp	7 mm	
			*Eromonas hydrophila*	1 mm	
	*Cinnamomum osmophloeum* leaves/EOs	Microdilution	*Escherichia coli*	IC_50_ = 250 μg/ml	Chang et al. (2001), Chang et al. (2008)
			*Enterococcus faecalis*	IC_50_ = 250 μg/ml	
			*Klebsiella pneumoniae*	IC_50_ = 500 μg/ml	
			*Salmonella* sp	IC_50_ = 500 μg/ml	
			*Vibrio parahemolyticus*	IC_50_ = 250 μg/ml	
			*Staphylococcus epidermidis*	IC_50_ = 250 μg/ml	
			MRSA	IC_50_ = 250 μg/ml	
			*Legionella pneumophila*	IC_50_ = 1,000 μg/ml	
	*Cinnamomum porrectum* stem bark/VO	Disk diffusion	MRSA	7.5 mm	Buru et al. (2014)
		Microdilution		IC_50_ = 500 μg/ml	
	*Cinnamomum tamala* stem bark/methanolic extract	Agar well diffusion	*Escherichia coli*	Without inhibition	Goyal et al. (2009)
			*Salmonella typhi*	11 mm	
			*Bacillus cereus*	14 mm	
			*Bacillus subtilis*	14 mm	
			*Staphylococcus aureus*	20 mm	
			*Streptococcus pyogenes*	13.5 mm	
			*Staphylococcus aureus*	IC_50_ = 256 μg/ml	
			*Streptococcus pyogenes*	IC_50_ = 4,096 μg/ml	
			*Bacillus subtilis*	IC_50_ = 4,096 μg/ml	
	*Cinnamomum verum* bark/EOs	Microdilution	*Staphylococcus aureus*	IC_50_ = 0.55 mg/ml	Unlu et al. (2010)
			*Streptococcus pyogenes*	IC_50_ = 0.55 mg/ml	
			*Streptococcus pneumoniae*	IC_50_ < 0.04 mg/ml	
			*Enterococcus faecalis*	IC_50_ = 1.15 mg/ml	
			*Enterococcus faecium*	IC_50_ = 1.12 mg/ml	
			*Bacillus cereus*	IC_50_ = 0.56 mg/ml	
			*Acinetobacter lwoffii*	IC_50_ < 0.04 mg/ml	
			*Enterobacter erogenes*	IC_50_ = 0.56 mg/ml	
			*Escherichia coli*	IC_50_ = 1.12 mg/ml	
			*Klebsiella pneumoniae*	IC_50_ = 0.14 mg/ml	
			*Proteus mirabilis*	IC_50_ = 0.14 mg/ml	
			*Pseudomonas eruginosa*	IC_50_ = 0.28 mg/ml	
			*Salmonella typhimurium*	IC_50_ = 0.14 mg/ml	
			*Mycobacterium smegmatis*	IC_50_ = 0.07 mg/ml	
			*Clostridium perfringens*	IC_50_ = 0.14 mg/ml	
			*Listeria ivanovii*	IC_50_ = 0.56 mg/ml	
			*Listeria innocua*	IC_50_ = 0.28 mg/ml	
			*Listeria welshimeri*	IC_50_ = 0.56 mg/ml	
			*Listeria seeligeri*	IC_50_ = 0.56 mg/ml	
	*Cinnamomum verum* bark/aqueous	Disk diffusion	*Moraxella cattarhalis*	11 mm	Rasheed and Thajuddin. (2011)
		Microdilution	*Moraxella cattarhalis*	IC_50_ = 120 mg/ml	
	*Cinnamomum verum* bark/EOs	Microdilution	*Pseudomonas eruginosa*	IC_50_ = 0.1125 mg/ml	Utchariyakiat et al. (2016)
	*Cinnamomum verum* bark/methanolic	Disk diffusion	*Proteus mirabilis*	5 mm	Hameed et al., (2016)
			*Pseudomonas eurogenosa*	4 mm	
			*Escherichia coli*	5.4 mm	
			*Klebsiella pneumonia*	6 mm	
			*Staphylococcus aureus*	5.2 mm	
	*Cinnamomum verum* bark/EOs	Microdilution	*Acinetobacter* spp.	IC_50_ = 625 μg/ml	Guerra et al., (2012), Noudeh et al., (2010)
			*Staphyllococcus aureus*	IC_50_ = 0.2 mg/ml	
			*Bacillus subtilis*	IC_50_ = 0.4 mg/ml	
			*Escherichia coli*	IC_50_ = 0.1 mg/ml	
			*Pseudomonas eruginosa*	IC_50_ = 0.2 mg/ml	
			*Agrobacterium tumefaciens*	IC_50_ = 12.5 mg/ml	
		Disk diffusion	*Staphylococcus aureus*	17.2 mm	Al-Bayati and Mohammed, (2009)
			*Bacillus cereus*	18.3 mm	
			*Escherichia coli*	15.7 mm	
			*Proteus mirabilis*	15.2 mm	
			*Klebsiella pneumonia*	17.5 mm	
			*Pseudomonas eruginosa*	14.4 mm	
			*Staphylococcus aureus*	IC_50_ = 62.5 μg/ml	
			*Bacillus cereus*	IC_50_ = 1.2 μg/ml	
			*Escherichia coli*	IC_50_ = 62.5 μg/ml	
			*Proteus mirabilis*	IC_50_ = 125.0 μg/ml	
			*Klebsiella pneumonia*	MIC = 62.5 μg/ml	
			*Pseudomonas eruginosa*	MIC = 125.0 μg/ml	
		Membrane permeability reduction test	*Escherichia coli*	↓wall cell permeability	Yap et al. (2015)
		Microdilution	*Pseudomonas putida*	IC_50_ = 1 mg/ml	Oussalah et al. (2006)
**Antidiabetic**	***In vitro* studies**				
	*Cinnamomum verum* bark/aqueous	α-amylase, *a*-glucosidase inhibition		IC_50_ = 0.5, 1.25, 2.5 mg/ml	Ranilla et al. (2010)
				↓α-amylase, ↓α-glucosidase	
	*Cinnamomum verum* bark/methanol	Yeast *a*-glucosidase, rat-intestinal *a*-glucosidase inhibition		IC_50_ = 5.83 μg/ml	Shihabudeen et al. (2011)
				↓yeast *a*-glucosidase	
				IC_50_ = 670 μg/ml	
				↓mammalian *a*-glucosidase	
	*Cinnamomum cassia* (L.) J. Presl/Bark/acetone and aqueous	Glucosidase, sucrase, maltase inhibition		↓α-glucosidase inhibitory activity ↑ sucrase and maltase inhibition	Kang et al. (2014)
	*Cinnamomum osmophloeum* twig/aqueous	PTP1B, *a*-glucosidase,α-amylase inhibition		↓ *a*-amylase, ↓α-glucosidase ↓ PTP1B	Lin et al. (2016)
	***In vivo* studies**				
	*Cinnamomum burmanni* (nees and T. Nees) blume/bark/aqueous	Rats/very high fat diet induced hyperglycemia 500, 300 mg/b.w.; oral		↓FBG, dose dependent manner	Cheng et al. (2012)
	*Cinnamomum cassia* (L.) J. Presl/bark/aqueous	Rats/glucose 2 g/kg b.w. i*p*; 85.7 mg/b.w oral		↓blood glucose control: Glibenclamide	Verspohl et al. (2005)
	*Cinnamomum cassia* (L.) J.Presl/aqueous	Mice/STZ induced diabetes; 100 mg/kg/day; oral		↓blood glucose	Chen et al. (2013)
				↑insulin	
	*Cinnamomum cassia* (L.) J.Presl/Bark/Methanol	Mice/STZ induced diabetes; 200 mg/b.w.; oral		↓ blood glucose	Kim et al. (2006)
	*Cinnamomum cassia* (L.) J. Presl bark/aqueous	Rats/alloxan induced diabetes 60 mg/b.w.; oral		↓blood glucose	Kamble and Rambhimaiah, (2015)
	*Cinnamomum porrectum* bark/polyphenols rich extract	Rats/STZ induced diabetes 100,200,300 mg/b.w.; oral		↓blood glucose	Jia et al. (2009)
	*Cinnamomum tamala*/leaves/ethanol	Rats/alloxan induced diabetes; 500 mg/b.w.; oral		↓ blood glucose	Kar et al. (2003)
	*Cinnamomum tamala* leaves/essential oil	Rats/STZ induced diabetes 10, 200 mg/b.w.; oral		↓ blood glucose	Kumar et al. (2012)
	*Cinnamomum verum* bark/water-soluble polyphenols	Rats/STZ induced diabetes 200 mg/kg, oral		↑weight loss ↓FBG, ↓PPG	Krishnakumar et al. (2014)
	*Cinnamomum verum* bark/volatile oil	Rats/STZ induced diabetes 20 mg/kg; oral		↓plasma glucose	Subash Babu et al. (2007)
	*Cinnamomum verum* essential oil	Rats/STZ induced diabetes 20 mg/kg; oral		↓blood glucose	Al-LogmaniI and Zari, (2009)
	*Cinnamomum verum* stem bark/chloroform	Rats/STZ induced diabetes 20 mg/kg; oral		↑muscle glycogen	Anand et al. (2010)
				↑hepatic glycogen	
				↓FBG	
	*Cinnamomum verum* bark/volatile oil	Rats/alloxan induced diabetes 5, 10, 20 mg/kg; oral		↓FBG, dose-dependent manner	Mishra et al. (2010)
				↓cholesterol	
				↓urinary protein	
				↓TBARS	
				↓blood urea	
				↓catalase	
	*Cinnamomum verum* bark/volatile oil	Rats/alloxan induced diabetes		↓FBG, dose-dependent manner	Rajbir et al. (2009)
		5, 10 and 20 mg/kg; oral			
	*Cinnamomumverum* sticks/Aqueous	Rats/STZ induced diabetes		↓blood glucose, dose-dependent manner	Shen et al. (2010)
		3, 30 and 100 mg/kg; oral			
	*Cinnamomumverum* bark/aqueous	Rats/oral glucose tolerance test		↓glycemic levels	Kannappan et al. (2006)
		0.2 ml day/rat; oral			
	*Cinnamomum verum*/bark/methanol	Rats/STZ induced diabetes		↓ postprandial hyperglycemia	Shihabudeen et al. (2011)
		300 mg/kg; oral			
**Anti-inflammatory**	***In vitro* studies**				
	*Cinnamomum cassia*/cinnamic aldehyde	RAW 264.7, LPS stimulated mice macrophages		IC_50=_ 0, 6.25, 12.5, 25, 50 μM anti-inflammatory	Liao et al. (2012a)
	*Cinnamomum camphora*/total crude extract/80% methanol, hexane, ethyl acetate fractions	RAW 264.7, LPS stimulated mice macrophages		IC_50=_ 100 μg/ml anti-inflammatory	Lee et al. (2006)
	***In vivo* studies**				
	*Cinnamomum cassia* bark oil/cinnamaldehyde	Rats		dose = 2–6 mg/kg bw	Chua et al. (2008)
				↓NF-kB	
	*Cinnamomum cassia* cinnamic aldehyde	Mice/carrageenan induced paw edema		dose = 1.25, 2.5, 5 mg/kg/bw	Liao et al. (2012a)
				Anti-inflammatory	
**Anti-cancer**	***In vitro* studies**				
	*C. burmanni* stem bark/methanolic	NPC/HK1, C666–1, human cancer cell lines		↑cytotoxicity	Daker et al. (2013)
				IC_50_ *=* 224.3 μg/ml	
				IC_50_ *=* 6.30 μg/ml	
	*C. cassia* bark aqueous	SiHa, human cervical carcinoma cell lines		↓growth of cancer cells	Koppikar et al. (2010)
				↑cytotoxicity	
				IC_50_ *=* 80 μg/ml	
	*C. cassia* ethanolic extract	HT 29, HCT 116, human colorectal carcinoma cell lines		↑Nrf2	Wondrak et al. (2010)
		↑antioxidant			
	*C. burmann* stem bark/aqueous	Lymphoma, melanoma, mice cancer cell lines		↓tumor cell growth	Kwon et al. (2010)
				↑cytotoxicity	
				IC_50_ = 0.5 mg/ml	
	*Cinnamomum* species cinnamaldehyde	Hep G2, hepatoma cells line		↑apoptosis	Ng and Wu. (2011)
				↑p53, ↑APO-1	
				↑cytotoxicity	
				IC_50_ = 9.8 μM	
	*C. subavenium* miq subamolide D subamolide E	SW 480, human colon adenocarcinoma cell lines A431, SCC1 human epidermoid carcinoma cells A375, human melanoma cell lines		↑DNA damage	Kuo et al. (2008); Yang et al. (2013)
				↑cytotoxicity	
				IC_50_ = 9.12 μg/ml	
				IC_50_ = 13.30 μg/ml	
				IC_50_ = 17.59 μg/ml	
	*C. subavenium* miq subamolide B, A	NTUB1, human urothelial carcinoma cell line SW480, human colon adenocarcinoma cell line		↓tyrosinase	Chen et al. (2007); Wang et al. (2011)
				↑apoptosis	
	*C. tenuifolium*/butanolides	DU145, human prostate cancer cell line		↓mitochondrial transmembrane potential	Lin et al. (2009)
				↑cytochrome C	
				↑caspase-9/caspase-3 ↑cytotoxicity	
**Neuroprotective**	***In vitro* studies**				
	*Cinnamomum* species water extract/procyanidin type a trimer	C6 glial cells, OGD exposed		↓glial cell swelling	Panickar et al. (2012)
				↓glutamate uptake	
	*Cinnamomum cassia*/extract/cinnamaldehyde	BV2 microglias, LPS activated		↓neuroinflammation	Ho et al. (2013)
				IC_50_ = 50 μg/ml	
	***In vivo* studies**				
	*Cinnamomum* species *trans*-cinnamaldehyde	Mice/6-OHDA treated intracerebroventricular		Anti-neuroinflammatory	Pyo et al. (2013)
				Dose = 30 mg/kg	
	*Cinnamomum zeylanicum* bark extract	Rats/SCOP treated intravenous		↑cognition dose = 100, 200, 400 mg/kg	Jain et al. (2015)
**Others pharmacological activities**	***In vivo* studies**				
	*Cinnamomum zeylanicum*/stem bark/methanol extract	Rats/l-name-induced hypertension, intravenous		Antihypertensive	Nyadjeu et al. (2011)
				dose = 5, 10, 20 mg/kg	
	*Cinnamomum zeylanicum*/bark and leaf/EOs	*Culex quinquefasciatus, Anopheles tessellatus* and *Aedes egypti*		Mosquitocidal	Samarasekera et al. (2005)
				Bark oil	
				*A. essellatus*	
				LD_50_ = 0.33 μg/ml	
				*C. uinquefasciatus*	
				LD_50_ = 0.66 μg/ml leaf oil	
				LD_50_ = 1.03–2.1 μg/ml	
	*Cinnamomum zeylanicum* bark/EOs	*Pediculushumanus capitis*		Ovicidal, adulticidal activities	Yang et al. (2005)
				LD_50_ = 0.5 mg/cm^2^	
	*Cinnamomum zeylanicum* bark/aqueous suspension	Rats		Anti-secretagogue	Alqasoumi. (2012)
				Antiulcer	
				dose = 250, 500 mg/kg b.w	
	*Cinnamomum zeylanicum* bark/ethanol extract	Rats		Pro-healing effect	Kamath et al. (2003); Farahpour and Habibi. (2012)
				dose = 250, 500 mg/kg b.w	
	*Cinnamomum zeylanicum* bark/ethanol extract	Rats/CCl_4_-induced liver injury		↑hepatoprotective	Eidi et al. (2012)
				Dose = 0.01, 0.05, 0.1 g/kg	

Abbreviations and symbols: ↑, increase; ↓, decrease; APOA-1, Apolipoprotein A-1; bw, body weight; FBG, fasting blood glucose; L-NAME, N(G)-nitro-L-arginine-methyl ester, LPS, lipopolysaccharide; p53, tumorprotein p53; PPG, postprandial plasma glucose; PTP1B, protein-tyrosine phosphatase; NF-κB, nuclear factor κB; OGD, oxygen-glucose deprivation; 6-OHDA, 6-hydroxydopamine; SCOP, scopolamine; STZ, streptozotocin; TBARS, thiobarbituric acid reactive substances.

Regarding the EO of *C. osmophloeum* leaves, the MIC values were 200 and 500 *μ*g/ml for the 9 bacteria tested (Chang et al., 2001), and the chemical components of this oil were similar to those of *Cinnamomum cassia* (L.) J. Presl (Hu et al., 1985). In addition, (Oussalah et al., 2006) evaluated the antibacterial efficacy of essential oils of two *Cinnamomum* species: *C. cassi* and *C. verum* against *Pseudomonas putida* and found MIC values of 0.05 and 0.1 % wt/vol, respectively. In another study, the MIC values for the EO of *C. cassia* leaves were 500 ppm for *E. coli*, *B. cereus*, *Lactobacillus sakei* and 750 mg/ml for *Salmonella typhimurium* (Turgis et al., 2012).

The antibacterial potency of the methanol extract of *C. cassia* was also tested against different species of *E. coli* by the disk diffusion method and the microdilution (Kim et al., 2004). The *C. cassia* bark EO was tested on *L. monocytogenes* and *EPEC* with a zone of inhibition of 22.42 and 13.72 mm, respectively, and a MIC of 0.03 and 0.06 μg/ml respectively (de Oliveira et al., 2012). Another study on the same part of *C. cassia* investigated the permeability of the bacterial membrane by measuring the relative electrical conductivity, which increased with the increased concentration of the EO (Huang et al., 2014).

The antimicrobial property of *C. verum* EO was assayed against 21 bacteria strains, using MIC methods and disc diffusion. The EO highlighted the significant antibacterial activity against the tested strains. It showed higher activity against Gram-positive (*Enterococcus*, *Streptococcus*, *Staphylococcus*) and Gram-negative (*Pseudomonas aeruginosa*) bacteria strains. These results are consistent with those of Chao et al. (2000) revealing that bark oil of cinnamon completely inhibited the growth of selected Gram-negative and Gram-positive bacteria.

*In vitro* antibacterial efficacy of different EOs (Al-Bayati and Mohammed, 2009; Noudeh et al., 2010; Guerra et al., 2012; Utchariyakiat et al., 2016) and methanol extracts (Hameed et al., 2016) of *C. verum* bark was evaluated against Gram-positive and Gram-negative bacteria using broth dilution and diffusion methods. The obtained results showed that *C. verum* was able to inhibit all of the tested strains. Furthermore, the main chemical constituents of *C. cassia* and *C. verum* natural oils were eugenol cinnamaldehyde (Ross, 1976; Hu et al., 1985) which could inhibit microbial growth (Lee and Ahn, 1998; Ooi et al., 2006).

EOs of *Cinnamomum micranthum* f. kanehirae (Hayata) S. S. Ying (leaves and twigs) were effective against *Vibrio parahemolyticus*, *V. alginolyticus*, *V. vulnificus*, *Lactococcus garvieae*, *Debaryomyces hansenii*, *Photobacteria damsel*, *Streptococcus* sp. and *Aeromonas hydrophila*, with diameters reduction from 0.1 to 1 cm (Yeh et al., 2009). The observed difference may be due to the difference of antibacterial constituents in leaves and twigs. The antibacterial screening of EO samples extracted from *Cinnamomum bejolghota* (Buch.-Ham). Sweet was evaluated using disc diffusion assay against zoonotic enteropathogens and showed promising antibacterial activity (Wannissorn et al., 2005). Indeed, EOs are complexes of antibacterial agents including natural aromatic and volatile compounds (Loy et al., 2001).

The volatile oils and extracts from the stem bark of *Cinnamomum impressicostatum* Kosterm., *C. altissimum* and *C. porrectum* (Roxb.) Kosterm showed high antibacterial activity against Gram-positive and Gram-negative bacteria, including methicillin-resistant *Staphylococcus aureus* (MRSA). Some tested extracts exhibited relevant activity against MRSA compared with methicillin-sensitive *S. aureus* (MSSA). The highest inhibition zone (21.0 mm) was measured for *C. impressicostatum* stem-bark water extract against MRSA with MIC and MBC values of 19.5 and 39.0 μg/ml, respectively (Buru et al., 2014). The leaf oil of *Cinnamomum chemungianum* M. Mohanan and A. N. Henry enhanced a moderate activity against the tested bacteria (Rameshkumar et al., 2007).

Agarwal et al. (2019) used *C. tamala* leaf extract to synthesize eco-friendly zinc oxide nanoparticles with important antibacterial effects against *S. aureus.* Authors showed that these nanoparticles induced reduction in bacterial growth, in a time and concentration-dependent pattern, due to membrane damage that leads to leakage of intracellular proteins and cellular contents. On the other hand, *C. verum* EOs exhibited remarkable inhibitory effects of *Staphylococcus aureus* multi-drug resistant (MDR). Indeed, cinnamon oil at concentrations of 3.12% strongly inhibited all the *Streptococcus mutans* isolated with the MIC values of 12.8–51.2 and 64–256 mg/ml, respectively (Fani and Kohanteb, 2011). (Rossi et al., 2019) showed that *C. verum* EOs inhibited the adhesion of *Salmonella enterica* which involved the reduction of polyphenol oxidase activity. (Wu et al., 2019) showed that *C. camphora* EOs had an important antibacterial potency against *E. coli* with a MIC and MBC of 200 μg/L.

(Yap et al., 2015) have studied the EO of *C. verum* bark against *E. coli* by testing the permeability of its outer membrane, and they found that the observed membrane damage was determined by denaturation and acidification of the cell membrane (Borges et al., 2013). This suggested that the EO of *C. verum* bark disrupts the cell membrane at lethal and sub-lethal concentrations by increasing the availability of the antibiotic in the bacterial cell (Eumkeb et al., 2012). Indeed, EOs are complexes of antibacterial agents including natural aromatic and volatile compounds (Loy et al., 2001).

### Antidiabetic Activities

Diabetes mellitus is a chronic disease that affects about 7% of the world's people (Zimmet et al., 2001) and it is expected to increase by 5.5% in 2025 (King et al., 1998). Diabetes mellitus type 2 (T2DM) accounts for 85–90% of all diagnosed diabetic patients (Wild et al., 2004) with high medical and social costs. Diabetes mellitus is characterized by an altered metabolism (of carbohydrates, lipids, and lipoproteins) and chronic hyperglycemia resulting from pancreatic β-cell dysfunction (Wild et al., 2004; Qin et al., 2010), insulin production deficiency, insulin resistance in key target tissues and impaired glycemic index control (Leiter and Lewanczuk, 2005), (DeFronzo et al., 1992). These alterations cause severe complications in the functioning of the cardiovascular system, as well as hypertension and dyslipidemia that are risk factors for stroke and myocardial infarction (Luscher and Steffel, 2008). Postprandial glucose control is essential for treating diabetes mellitus and avoiding its complications (Ortiz-Andrade et al., 2007). Moreover, correct diet and sport are necessary for the prevention of T2DM (Kahn et al., 2006; Hu, 2011).

**FIGURE 3:**
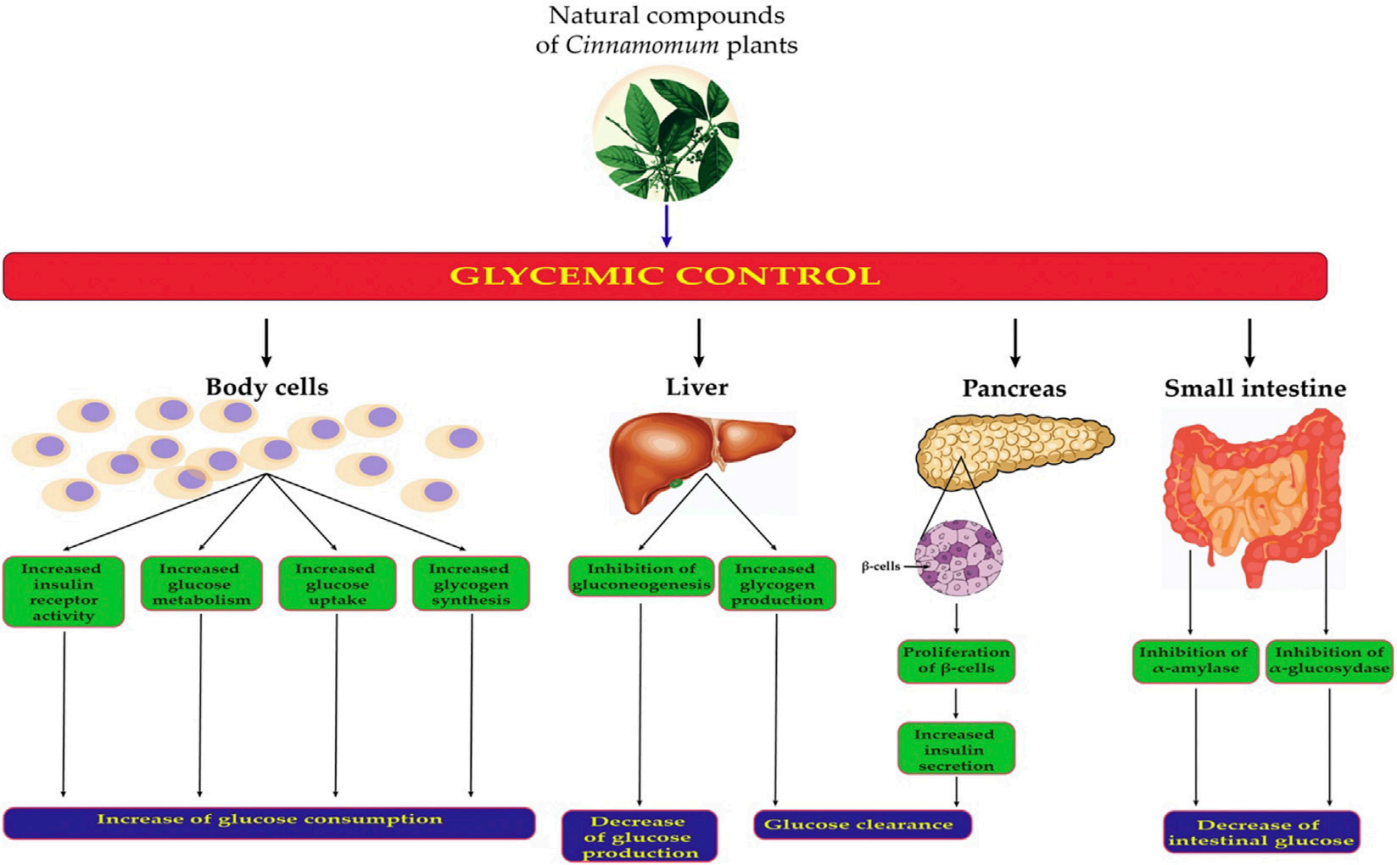
Potential mechanisms of antidiabetic effects of chemical compounds of *Cinnamomum* plants. Body cells mean all the human cells with receptors for insulin.

The antidiabetic mechanisms of natural compounds derived from *Cinnamomum* spp. are explained as follow: 1) stimulation of insulin secretion by pancreatic β-cells of the islets of Langerhans, 2) increasing the muscle and hepatic glycogen content, 3) inhibition of α-amylase and α-glucosidase activities (key enzymes of carbohydrate metabolism) (Subash Babu et al., 2007; Zhang et al., 2008) 4) stimulation of glycogen synthesis and cellular glucose uptake by membrane translocation of glucose transporter (GLUT 4); stimulation of the glucose metabolism, 5) reduction of the gluconeogenesis by its actions on the most important regulatory enzymes, 6) potentiation of insulin release and increasing insulin receptor activity (Bandara et al., 2012; Ranasinghe et al., 2012). (Figure 3 **)** and Table 2.

The antihyperglycemic action of the soluble polyphenols of *C. verum* bark was verified in SZT-induced diabetic rats at the dose of 200 mg/body weight (Krishnakumar et al., 2014). Cinnamaldehyde isolated from the volatile oil of *C. verum* showed a highly significant effect on plasma glucose levels using a rat model of SZT-induced diabetes (Subash Babu et al., 2007). This major component has a wide variety of pharmacological properties, including antihyperglycaemic activity in diabetic rats (Subash Babu et al., 2007; Zhang et al., 2008). Al-LogmaniI and Zari (2009) have also tested the EO of *C. verum* and showed an important reduction in blood sugar levels.

The chloroform extract of *C. verum* bark stem exhibited antidiabetic activity *in vivo* at 20 mg/body weight using a rat model of SZT-induced diabetes (Anand et al., 2010). The authors demonstrated that the chemical compounds of extract increased the muscle and liver glycogen content. By using alloxan-induced diabetes in rats, other studies showed that the EO of *C. verum* bark decreased fasting blood sugar in a dose-dependent manner and reduced total cholesterol level, blood urea, urinary protein, thiobarbituric acid reactive substances (TBARS), and catalase levels in diabetic rats (Rajbir et al., 2009; Mishra et al., 2010). A few studies have reported the antidiabetic effect of *C. verum* aqueous extracts (Verspohl et al., 2005; Kannappan et al., 2006; Ranilla et al., 2010; Shen et al., 2010; Chen et al., 2013).

The antihyperglycemic activity can also be evaluated by the inhibition test of α-glucosidase and α-amylase. Inhibitors of these enzymes are intended to maintain glucose homeostasis in diabetics by decreasing the rate of glucose uptake (Bösenberg and van Zyl, 2008; Hanhineva et al., 2010). (Ranilla et al., 2010) showed an important inhibition of α-glucosidase and α-amylase actions by *C. verum* bark aqueous extract. The same extract attenuated hyperglycemia depending on the dose in SZT diabetic rats (Shen et al., 2010; Chen et al., 2013). The antidiabetic effect of this extract was confirmed by the decrease in blood glucose using oral glucose tolerance test in rats (Kannappan et al., 2006; Chen et al., 2013).

In an *in vitro* study, *C. verum* showed a potential antidiabetic effect by reducing post-prandial intestinal glucose absorption via enzymatic reduction of pancreatic α–amylase and intestinal α–glucosidase. *In vivo* studies also confirmed the anti-hyperglycemic effects of *C. verum* (Bandara et al., 2012; Ranasinghe et al., 2012) through the decrease in LDL cholesterol, total cholesterol and triglycerides with increasing HDL in hyper-lipidaemic albino rabbits. (Javed et al., 2012).

### Antioxidant Activity

Some clinical investigations showed that long term consumption of cinnamon extracts improved the levels of blood markers of oxidative stress, such as the antioxidant capacity. The extracts also reduced the transaminase and lipid peroxidase activities (Ranjbar et al., 2007; Rashidi et al., 2014). Diverse tests were used to evaluate potential antioxidant action of *Cinnamomum* plant extracts and secondary metabolites. These include 2,2′-azinobis (3-ethyl-benzothiazoline-6-sulphonic acid (ABTS), 2,2-diphenyl-1-picrylhydrazyl (DPPH), ferric reducing antioxidant power (FRAP), oxygen radical absorbance capacity (ORAC), Folin Ciocalteau reduction assay (FCR), β carotene linoleic acid bleaching (BCLB), and enzyme inhibition assays.

The antioxidant activity of *C. osmophloeum* leaf EOs was assessed using DPPH radical scavenging assay, with IC_50_ = 29.7 μg/ml (Lin et al., 2007). The ethanol extract of the same species was tested using DPPH and FRAP assays by (Lee et al., 2015). The results showed inhibitory values of 38.97 and 0.48% by DPPH and FRAP test, respectively.

(Prasad et al., 2009) tested the antioxidant activity of ethanolic extract of *Cinnamomum curvifolium* (Lam.) Nees, *C. burmanni, C. cassia, C. verum,* and *C. tamala* using DPPH, FRAP and ORAC assays. All tested species showed important antioxidant activities with some variability depending on the species and the used method (Prasad et al., 2009). *C. cassia* was also reported as an antioxidant species by several studies (Lin et al., 2003; Kim et al., 2006; Jang et al., 2007). *C. cassia* water extract showed important capacities to inhibit the key enzymes involved in ROS generation such as catalase, superoxide dismutase and glutathione peroxidase (Kim et al., 2006). Methanolic extract of *C. verum* revealed remarkable antioxidant activities particularly by chelating metal and inhibition of lipid peroxidation (Mathew and Abraham, 2006). Moreover, the inhibition of lipid peroxidation was also demonstrated by EOs of *C. verum* (Jayaprakasha et al., 2003; Jayaprakasha et al., 2006). The research that highlighted the antioxidative activity of *Cinnamomum* plants is summarized in Table 2.

### Anti-inflammatory Activities

Inflammation is an important pathophysiological process of the organism that maintains homeostasis, fighting pathogens and repairing damaged tissues caused by various injuries such as trauma, infection or immune response (Salehi et al., 2019b), (Stalnikowitz and Weissbrod, 2003). The same inflammatory process is involved in the maintenance of several disorders and is characterized by the production of diverse pro-inflammatory mediators (Salehi et al., 2020a). The current common class of medications against inflammation disorders still relies on non-steroidal anti-inflammatory drugs (Padureanu et al., 2019). However, in spite of having important anti-inflammatory potential, these drug class can cause several side effects such as bleeding, kidney failure and gastrointestinal ulceration. Therefore, increasing attention has been directed towards natural and health-friendly alternatives (Salehi et al., 2019c; Salehi et al., 2019d). The use of natural compounds constitutes an attractive approach for the treatment of several inflammatory disorders.

Inflammation involves reactive oxygen species (ROS) generation, NO production, phospholipase A_2_ activation and histamine release in neutrophils, macrophages and mast cells. NO has an important role in lipopolysaccharide (LPS), TNF or IL-1 mediated inflammatory process. It is also essential in the cell function maintenance (Stalnikowitz and Weissbrod, 2003) though, on the other hand, NO is able to induce cell injury as a reactive radical. The activator protein-1 (AP-1) and nuclear factor kappa B (NF-kB) are important modulators of inflammation.

The main mechanism of the anti-inflammatory activity of the major chemical compound of *Cinnamomum* plants, cinnamaldehyde is the effective inhibitor of the expression of NF-kB mediated by its antioxidant activity (Kim et al., 2007). In addition, cinnamaldehyde inhibits pro-inflammatory mediators such as chemokines, interferons, interleukins, lymphokines, eicosanoids (prostaglandins and leukotrienes) and ROS involved in Alzheimer’s disease (Latta et al., 2015). Preclinical studies showed a reasonably good anti-inflammatory effect of *Cinnamomum* constituents. Diverse extracts and isolated compounds of *Cinnamomum* plants have been studied for possible anti-inflammatory activity in various animal models. (Liao et al., 2012b) showed an anti-inflammatory effect of cinnamaldehyde on lipopolysaccharide (LPS) stimulated mouse macrophage (RAW 264.7) at 50 µM.

The *in vivo* investigations confirmed the anti-inflammatory effect using the carrageenan-induced mouse paw edema (Liao et al., 2012b). Cinnamic alcohol (another volatile compound from *Cinnamomum* plants) also exerted anti-inflammatory activity using the same model (Liao et al., 2012b). In another study, cinnamyl acetate revealed a significant anti-inflammatory action on LPS-stimulated mouse macrophages (RAW264.7) (Chua et al., 2008). The research highlighting the anti-inflammatory activity of *Cinnamomum* plants is displayed in Table 2.

### Anticancer Activities

Recent researches showed that the *in vivo* anticancer activity of the cinnamon extract was mediated by the induction of tumor apoptosis through the inhibition of NF-κB levels (Kwon et al., 2010). On the other hand, cinnamon showed important anti-cancer effects via affecting on numerous cancer-related pathways such as apoptosis (Kwon et al., 2010). This apoptotic action is mediated by targeting Fas/Fas/CD95, caspase-3, and Bcl-XL (B-cell lymphoma-extra-large) pathways (Sadeghi et al., 2019).

C. burmanni stem bark methanolic extract was tested on human cell lines (NPC/HK1 and C666-1) by Daker et al. (2013). The results showed important cytotoxic effect against HK1 (IC50 = 224.3 μg/ml) and C666-1 (IC50 = 6.30 μg/ml) Daker et al. (2013). Koppikar et al. (2010) tested the aqueous bark extract of C. cassia on human cervical carcinoma (SiHa) cell lines. This extract decreased the growth of cancer up to 2-fold compared to the untreated control cells at the concentration of 80 μg/ml Koppikar et al. (2010).

In another study, the ethanolic extract of the same species tested by Wondrak et al. (2010) on human colorectal carcinoma (HCT 116 and HT 29) cell lines showed anticancer properties. The *C. cassia* bark aqueous extract was evaluated on cancer cell lines of lymphoma, melanoma and cervix as well as in a melanoma mouse model (Kwon et al., 2010). The cinnamon extract inhibited tumor cell growth *in vitro* at 0.5 mg/ml.

Cinnamaldehyde showed an important antitumor effect as well (Ng and Wu, 2011). It inhibited the growth of hepatoma Hep G2 cells line at IC_50_ = 9.8 μM. Some bioactive compounds isolated from *Cinnamomum subavenium* Miq. such as subamolide D and E showed remarkable anticancer effects on human colon adenocarcinoma (SW 480) cell lines. The cytotoxic effect was mediated by the capacity of these compounds to cause DNA damage in a dose- and time-dependent manner (Kuo et al., 2008). Moreover, subamolide B isolated from the stem of the same plant showed significant cytotoxic effects on human SCC12 (IC_50_ = 9.12 μg/ml), epidermoid carcinoma A431 (IC_50_ = 13.30 μg/ml), and human melanoma A375 (IC_50_ = 17.59 μg/ml) cell lines (Yang et al., 2013).

In another study, subamolide B and its isomer subamolide A induced apoptosis in human colon adenocarcinoma cell line SW480 and human urothelial carcinoma cell line NTUB1(Chen et al., 2007; Wang et al., 2011). Furthermore, subamolide E, isolated from *C. subavenium,* exhibited an important *in vitro* anti-melanoma activity (Kuo et al., 2008; Wang et al., 2011). (Lin et al., 2009) reported that butanolides isolated from the stem of *Cinnamomum tenuifolium* (Makino) Sugim showed anticancer activity on human prostate cancer (DU145) cell line. (Lin et al., 2009). In addition, the extracts of *C. kotoense* was found cytotoxic against HeLa cells (Chen et al., 2008). Butanolides isolated from the *C. kotoense* leaves reported genotoxic and cytotoxic effects on various cell lines, such as human laryngeal carcinoma cells Hep-2, Chinese hamster ovarian cells CHO-K1 and rat hepatoma tissue cultures (Garcez et al., 2005) and mouse lymphoid leukemia P-388 cells (Tsai et al., 2002). *In vitro* antineoplastic activities of *Cinnamomum* species are summarized in Table 2.

### Neuroprotective Activities: Potential Mechanisms and Molecular Targets in Neurodegenerative Diseases

**FIGURE 4:**
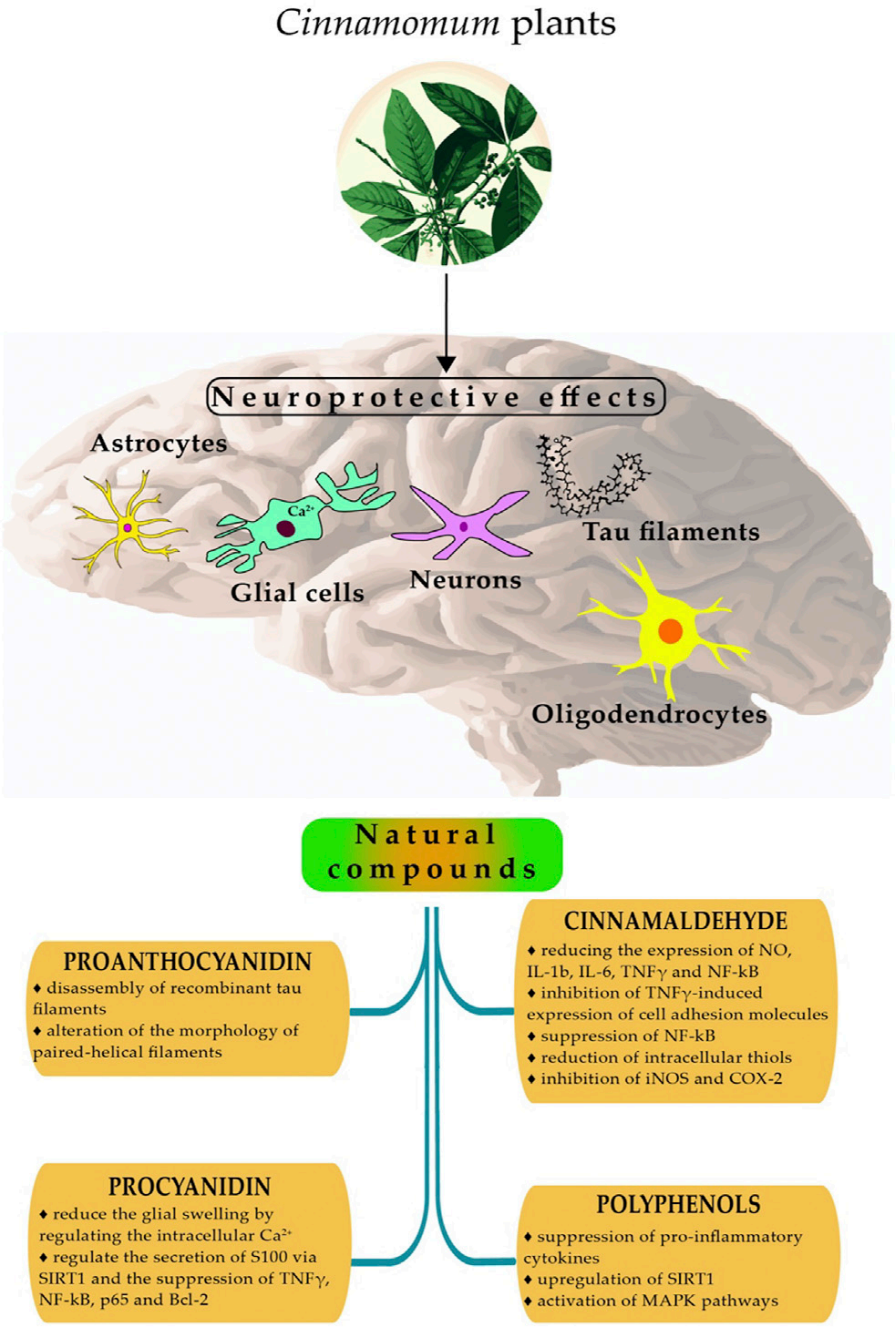
Summarized mechanisms of neuroprotective effects of Cinnamomum plants. Abbreviations: SIRT1, Sirtuin 1; MAPK, mitogen-activated protein kinase; iNOS, inducible Nitric Oxide synthase; TNF-γ, tumor necrosis factor; COX-2, Cyclooxygenase-2; NF-kB, Nuclear Factor- Kappa B; p65 (RelA subunit of NF-κB family of transcription factors); Bcl-2, B-cell lymphoma 2; IL-1b, Interleukin-1beta; IL-6, Interleukin 6.

Parkinson's and Alzheimer’s diseases are common neurodegenerative diseases, accompanied by cognitive and memory impairments*,* sometimes difficult to differentiate from real psychosis or other neurological diseases (Nussbaum et al., 2017; Buga et al., 2019; Tsatsakis et al., 2019). The mechanisms of neuroprotective effects of *Cinnamomum* plants and their derivatives have been reported by several studies (Liao et al., 2008; Peng et al., 2008; Peterson et al., 2009; Panickar et al., 2012; Jiao et al., 2013). (Figure 4).

(Ho et al., 2013) showed that procyanidinA trimer 1 (a bioactive compound isolated from *C. burmanni*) had an essential neuroprotective effect which reduced the glial swelling by regulating the intracellular calcium concentration in glial neuronal cells (Peng et al., 2008). Procyanidin exhibited neuroprotective activity by its capacity to regulate the secretion of S100 via the regulation of SIRT1 and the suppression of TNF-γ, NF-kB p65 (RelA subunit of NF-κB family of transcription factors) and B-cell lymphoma 2 (Bcl-2) on glioma cells (Jiao et al., 2013).

(Panickar et al., 2012) studied the neuroprotection of procyanidin B2 isolated from *C. verum.* The results showed that this molecule inhibited advanced glycation end-product production in the bovine serum albumin-glucose model (Panickar et al., 2012).

Cinnamaldehyde is a volatile compound found in *C. cassia* extracts. (Ho et al., 2013) showed that this compound possessed an important neuroprotective effect by reducing the expression of NO, tumor necrosis factor (NF-γ), interleukin-1beta (IL-1b), interleukin 6 (IL-6), and nuclear factor-kappa B (NF-kB) in LPS induced BV2 microglia cells.

In a recent study, (Liao et al., 2008) revealed that cinnamaldehyde from *C. cassia* exhibited remarkable neuroprotection by the reduction of TNFγ-induced expression of cell adhesion molecules, the suppression of nuclear factor-kappa B (NF-kB) and the reduction of intracellular thiols in endothelial cells (Liao et al., 2008).

Cinnamaldehyde isolated from *C. ramulus* exhibited anti-neuro-inflammatory properties by the inhibition of inducible nitric oxide synthase (iNOS) and cyclooxygenase-2 (COX-2) in LPS-induced BV2 microglial cells (Pyo et al., 2013).

In addition, cinnamon bioactive compounds exhibited neuroprotective effects in *in vivo* models of Alzheimer’s disease and extended the lifespan via regulation of key antioxidant pathways (Crews et al., 2016). *C. verum* extracts provided an important protection against Alzheimer’s disease and dementia in the scopolamine-induced memory impairment in experimental rat model (Jain et al., 2015).

In the same way, a different mixture of cinnamon rich in polyphenols exhibited important neuroprotective activities in rat glioma cells by suppression of pro-inflammatory cytokines, upregulation of sirtuin 1 (SIRT1) and activation of mitogen-activated protein kinase (MAPK) pathways (Qin et al., 2014). Neuroprotective activities of *Cinnamomum* plants are tabulated in Table 2.

### Other Pharmacological Activities

Cinnamomum plants have also shown an array of other biological activities. (Nyadjeu et al., 2011) reported that *C. verum* extracts decreased arterial blood pressure in rats with normal arterial hypertension and various models of hypertensive rats (salt-loaded and spontaneously) (Nyadjeu et al., 2011). In another study, (Wansi et al., 2007) reported that *C. verum* extracts showed similar effects in arterial blood pressure of normotensive rats and salt-loaded hypertensive rats. Moreover, they showed that *C. verum* had a vasorelaxant action on the thoracic aortic ring segments, which suggests that *C. verum* might inhibit extracellular calcium through L-type voltage-sensitive channels (Wansi et al., 2007).

The antiparasitic effects of EOs from bark and leaves of *C. verum* were investigated by (Samarasekera et al., 2005) against *Anopheles tessellates, Culex quinquefasciatus*, and *Aedes aegypti*. These oils showed knock-down and mortality against *A. tessellatus* (LD_50_ = 0.33 μg/ml) and *C. quinquefasciatus* (LD_50_ = 0.66 μg/ml). (Yang et al., 2005) showed fewer effect of *C. verum* bark EO against *Pediculus humanus capitis* (eggs, adult females of human head louse) than either phenothrin or pyrethrum using direct contact bioassay (Yang et al., 2005).

Collagen synthesis was stimulated in human dermal fibroblasts by *C. verum* extracts (Takasao et al., 2012). *C. verum* extract enhanced both mRNA and protein expression levels of type I collagen without cytotoxicity, and cinnamaldehyde was the most active component stimulating the expression of collagen, suggesting that *C. verum* extracts might be useful in skin anti-aging treatments (Takasao et al., 2012). Moreover, *C. verum* extracts inhibited osteoclastogenesis (Tsuji-Naito, 2008). At concentrations of 12.5–50 μg/ml, *C. verum* inhibited osteoclast-like cell formation in a dose-dependent manner without affecting cell viability. This finding suggests its potential use in the treatment of osteopenia diseases (Tsuji-Naito, 2008). In addition, *C. verum* exhibited *in vivo* anti-secretagogue and anti-gastric ulcer effects (Alqasoumi, 2012). In pylorus-ligated rats, *C. verum* extract pre-treatment reduced the basal gastric acid secretion and inhibited gastric hemorrhagic lesions (Alqasoumi, 2012). Furthermore, *C. verum* extracts at 100 and 200 mg/b. w. effectively diminished the extent of diarrhea (71.7 and 80.4%, respectively) in test animals (RAO, 2012).

In another research performed in two rat models, the evaluation of anti-nociceptive effects of *C. verum* and selected plants showed that *C. verum* produced a dose-dependent analgesic protective effect against thermal stimuli. Moreover, *C. verum* enhanced an anti-inflammatory activity against chronic inflammation of cotton pellet granuloma (Atta and Alkofahi, 1998; RAO, 2012). *C. verum*has also demonstrated wound healing properties (Kamath et al., 2003; Farahpour and Habibi, 2012). *C. verum* (0.01, 0.05, and 0.1 g/b. w. for 28 days) displayed hepatoprotective effects in a study where the liver injury was induced in rats by CCl_4_ (carbon tetrachloride) (Eidi et al., 2012). Other pharmacological properties of *Cinnamomum* plants are summarized in Table 2.

## Clinical Trials Related to Efficacy of Bioactive Compounds Derived From *Cinnamomum*

### Type 2 Diabetes Mellitus, Obesity and Metabolic Syndrome

Cinnamon consumption was associated with reduction in the levels of fasting plasma glucose (FPG), total cholesterol (TC), triglyceride levels (TG), LDL cholesterol and hemoglobin A1c (HbA1c). In one study, cinnamon consumption resulted in significantly increased FPG levels and HbA1c (Blevins et al., 2007) or no significant change in HDL cholesterol levels. However, the most preferred and effective doses with fewer side effects are still not clear. Doses applied in randomized clinical trials showed conflicting results as consumption of 1 g/day (Khan et al., 2003; Blevins et al., 2007; Crawford, 2009), 1.5 g/day (Vanschoonbeek et al., 2006; Radhia et al., 2010), 2 g/day (Akilen et al., 2010), 3 g/day (Khan et al., 2003; Mang et al., 2006; Vafa et al., 2012), or 5 g/day (Gutierrez et al., 2016), 6 g/day (Khan et al., 2003) showed different effects in glycemic and lipid parameters. FPG and HbA1c are commonly used parameters for diabetes diagnosis. HbA1c values of more than 6.5% can be related to diabetes. Vafa et al. showed FPG and HbA1c at baseline modestly elevated (7.02 mmol/L to 8 mmol/L) and 6.9–8.0%, respectively (Vafa et al., 2012). Both the studies by Akilen et al. and Crowfrod reported a drop of 0.36 and 0.83% of HbA1c values at low dose cinnamon supplementation which were safe and well tolerated for 3 months in patients using simultaneous hypoglycemic medications (Crawford, 2009; Akilen et al., 2010). Mang et al. observed that aqueous extract of cassia cinnamon significantly reduced glucose levels like 10.3% in the cinnamon group and 3.4% in the placebo group, however, no considerable difference in HbA1c and cholesterol (LDL, HDL, TC) levels were observed (Mang et al., 2006). Suppapitiporn et al. observed no significant differences in cinnamon and placebo group for FPG and lipid profiles but HbA1c level decreased in both groups by 0.38 and 0.19% respectively (Suppapitiporn and Kanpaksi, 2006).

Khan et al. reported that intake of 1, 3, or 6 g of cinnamon reduced fasting serum glucose by 18–29%, LDL by 7–27%, triacylglycerol by 23–30%, and total cholesterol by 12 26% after 40 days treatment. Nevertheless, they did not investigate the HbA1c value (Khan et al., 2003). One study showed that, consumption of *Cinnamomum cassia* powder 1.5 g/day for 6 weeks, did not improve plasma glucose levels, insulin, and cholesterol levels and no reduction in HbA1c level was noted (Vanschoonbeek et al., 2006). Only one trial by Blevins et al. observed significant increases in HbA1c and FPG levels (Blevins et al., 2007). In healthy, sedentary and obese women with *Cassia* supplementation a statistically significant reduction in glucose level was noted suggesting that the bark of *Cassia*can improve glucose tolerance. However, this study did not mention the acute dose of *Cassia* to maintain insulin resistance or sensitivity (Gutierrez et al., 2016). Some studies proposed that cinnamon also improved lipid profiles in clinical trials. Vafa et al. showed that in type 2 diabetes treated with cinnamon increased LDL levels but decreased TC, insulin and triglyceride levels with the improved glycemic index (Vafa et al., 2012). In addition, intercellular adhesion molecule-1 (ICAM-1) levels are increased in serum associated with increasing type-2 diabetes. Azimi et al. studied that consumption of *Cinnamomum verum* extract for 8 weeks decreased serum ICAM-1 level in patients with type-2 diabetes (Azimi et al., 2016).

26 clinical trial studies on cinnamon are present on Clinical Trials Govt. Database, among them most of the studies are under process and 14 studies are completed. Clinical trial numbers NCT03219411 and NCT01301521 showed effects of cinnamon supplementation in pre-diabetic patients. Clinical trials NCT03711682 and NCT00237640 observed plasma glucose and lipid levels reduction mediated by cinnamon in type 2 diabetic patients and in noninsulin dependent type 2 diabetes mellitus patients respectively. NCT01302743 trial demonstrated the application of water-soluble cinnamon bark extract and metformin for the treatment of type 2 diabetes mellitus patients. Table 3 displayed the clinical studies on Cinnamon plants in relation to diabetes.

**TABLE 3:** Description of recent clinical studies related to pharmacological activity of natural compounds from *Cinnamomum* species.

Pharmacological activity	Clinical trial/study design (type, patients included)	Period, country	Intervention (doses of *Cinnamon* and its derivatives)	Standard comparison	The most representative clinical outcomes	Ref
Type 2 diabetes	Two groups included: Cinnamon- group 1,2, 3	Department of Human Nutrition, NWFP Agricultural University, Peshawar, Pakistan	Cinnamon group: 500 mg capsule of *Cinnamomum cassia* placebo group: Wheat flour, 500 mg, 1 g or 2 capsules per day for 20 days; group 1 and 4: 3 g or 6 capsules per day for 20 days; group 2 and 5: 6 g or 12 capsules per day for 20 days; group 3 and 6; 6 g or 12 capsules per day for 40 days	Placebo	↓serum glucose (18–29%), ↓TG (23–30%), ↓LDL-C (7–27%) ↓total cholesterol (12–26%)	Khan et al. (2003)
	Placebo- group 4, 5, 6					
	Cinnamon group: 60 patients					
	Age 52.2 ± 6.32 years					
	Not on insulin therapy					
	Not taking other medicine					
	Fasting blood glucose					
	Levels 7.8–22.2 mmol/L					
Type 2 diabetes	Cinnamon group: 33 patients, age 62·8 ± 8·37	Hannover, Germany	Cinnamon group: Extract 112 mg aqueous cinnamon extract placebo: Microcrystalline cellulose 3 g powder per day, three times a day for 4 months	Placebo	↓glucose levels up to 10.3%	Mang et al. (2006)
	Body weight 88·5 ± 19·1 kg					
	HbA1c 6.7–6.9%					
	Placebo group: 32 patients age 63·7 ± 7·17 years					
	Body weight 89·9 ± 14·1 kg					
	HbA1c 6.7–6.9%					
Type 2 diabetes	Cinnamon group	Netherlands	Cinnamon group: 500 mg of *Cinnamomum cassia* placebo group: 500 mg, wheat flour, verstegen 1.5 g per day, 1 capsule at breakfast, lunch and dinner for 6 weeks	Placebo	↓plasma glucose, ↓plasma insulin, ↓total cholesterol, ↓LDL, ↓TG ↓HDL ↓HbA1c	Vanschoonbeek et al. (2006)
	12 postmenopausal women age 62 ± 2 years					
	Body weight 85.4 ± 3.6 kg					
	Placebo group: 13 patients					
	Age 64 ± 2 years					
	Body weight 82.2 ± 4.0 kg					
Type 2 diabetes	Cinnamon group: 30 patients	US	Cinnamon group: 500 mg capsule of *Cinnamomum cassia* placebo group: Wheat flour, 500 mg capsule: 1 g per day, for breakfast and dinner for 3 months	Placebo	No significant change in FBG, lipid for cinnamon group	Blevins et al. (2007)
	Age 63.6 years					
	Placebo: 22 patients					
	Age 58.0 years					
Type 2 diabetes	Cinnamon group: 55 patients	United States military base, May 2007 to August 2007	Cinnamon group: Capsules 500 mg capsule of *Cinnamomum cassia* control group: 1 g/day with food for 90 days	Control	Cinnamon group ↓ HbA1c	Crawford. (2009)
	Age 60.5 ± 10.7					
	HbA1c ≥ 7.0% control group: 54 patients					
	Age 59.9 ± 9.2 years					
	HbA1c ≥ 7.0%					
Multi-ethnic type 2 diabetic	Cinnamon group: 58 patients, age 54.9 ± 9.8 years	October 2007 to January 2009, United Kingdom	Cinnamon group: 500 mg capsule of *Cinnamomum cassia* placebo: Starch, 500 mg capsule 2 g per day, for breakfast (1 capsule), lunch (2 capsules), dinner (1 capsule) for 12 weeks	Placebo	↓HbA1c ↓FPG ↓BMI	Akilen et al. (2010)
	Body weight 74.94 ± 13.34; FPG≥ 7 mmol/L					
	HbA1c ≥ 7.0% placebo group: 22 patients					
	Age 55.67 ± 7.98 years					
	Body weight 73.02 ± 10.38 years FPG≥7 mmol⁄l					
	HbA1c ≥ 7.0%					
Type 2 diabetes	Cinnamon group: 22 patients	Tehran, Iran	Cinnamon group: 500 mg capsule of *Cinnamomum zeylanicum* placebo group: 500 mg capsule, wheat flour 3 g per day, 2 capsules at breakfast, lunch and dinner for 8 weeks	Placebo	No significant difference in cinnamon and placebo group on HbA1c, ↓TG, ↓Insulin ↑ LDL-C	Vafa et al. (2012)
	Age 54.11 ± 10.37 years					
	Body weight 74.94 ± 13.34					
	Placebo group: 22 patients					
	Age 55.67 ± 7.98 years					
	Body weight 73.02 ± 10.38 kg					
Type 2 diabetes	Cinnamon group: 137 patients	Beijing and dalian, China	Cinnamon group: Water extract of cinnamon and CinSulin^®^, 250 mg/capsule placebo: 500 mg capsule, wheat flour daily, twice a day for 2 months	Placebo	↓LDL-C ↓HDL ↓HOMA-IR	Anderson et al. (2015)
	Age 61.3 ± 0.8 years					
Type 2 diabetes	Cross-over study cinnamon group:10 sedentary obese females	Texas, United States	Cinnamon group: 1–6 g/day powder of *C.* *cassia/aromaticum* and *C.* *zeylanicum/verum* placebo: Cellulose powder 5 g	Placebo	No differences observed in blood glucose, serum insulin, insulin sensitivity, insulin resistance	Gutierrez et al. (2016)
	Age 22.7 ± 4 years					
	Body weight 104.42 ± 16.75 kg					
	Take oral/intrauterine contraceptives					
	Prescription medications					
	Over-the-counter weight loss pills					
Type 2 diabetes	Cinnamon group: 40 patients	September 2012 to December 2012, Iran	Cinnamon group*: Cinnamomum verum* 3 g for 8 weeks control group: 3 glasses of tea, for 8 weeks	Control	↓sICAM-1	Azimi et al. (2016)
	Age 54.15 ± 1.0 years					
	Weight 75.62 ± 1.2 kg					
	Control group:40 patients					
	Age 53.64 ± 1.3 years					
	Weight 78.74 ± 1.2 kg					
Polycystic ovary syndrome	Herbal medicine plus lifestyle intervention study	August 2012 to January 2014, Australia	*Glycyrrhiza glabra, Paeonia lactiflora, Cinnamomum verum, Hypericum perforatum:* three tablet per day for 3 months	Lifestyle intervention	↓ oligomenorrhoea ↓ BMI ↓ weight	Arentz et al. (2017)
	Cinnamon group: 60 overweight women					
	Age 29.2 ± 5.6 years					
	Weight 93.2 ± 18.9 kg					
	Lifestyle intervention group: 62 patients age 28.9 ± 5.6					
	Weight 97.3 ± 21.3 kg					
Polycystic ovary syndrome	Cinnamon group: 29 patients	Iran	Cinnamon group: 500 mg capsules (450 mg capsule of starch and 50 mg cinnamon powder): 1.5 g per day three times, after a meal with 10 mg medroxyprogesterone tablet from 15th day of menstruation cycle for 10 days for 12 weeks	Placebo	↓fasting insulin, ↓HOMA-IR, ↓LDL, ↓TG, ↓testosterone, ↓ insulin, ↓ weight ↓HbA1c	Hajimonfarednejad et al. (2018a)
	Age 18–45 years					
	Weight 68.24 ± 9.68 kg					
	Placebo group: 30 patients					
	Age 18–45 years					
	63.26 ± 11.62 kg					
Polycystic ovary syndrome	Cinnamon group: 42 patients with rotterdam criteria	september 2015 to januray 2016, tehran, Iran	Cinnamon group: 500 mg capsule/day placebo: Wheat flour: 1.5 g per day for 8 weeks	Placebo	↑antioxidant capacity ↓malondialdehyde↓ BMI	Borzoei et al. (2018a)
	Age 29.26 years					
	Placebo group: 42 patients with rotterdam criteria					
	Age 30 years					
Polycystic ovary syndrome	Cinnamon group: 42 patients with rotterdam criteria	Mohheb Yas Hospital, Tehran, Iran, October 2015 to February 2016	Cinnamon group: 500 mg capsule/day placebo: Wheat flour: 1.5 g per day for 8 weeks	Placebo	↓TG, ↓BMI, ↓TC ↓HOMA-IR ↓insulin, ↓ LDL-C HDL-C unchanged	Borzoei et al. (2018b)
	Age 29.3 years					
	Weight 76.6 kg					
	Placebo group: 42 patients with rotterdam criteria					
	Age 30.2 years					
	Weight 77.7 kg					
Polycystic ovarian syndrome	Cinnamon group	March 2011 to April 2014 to United States	Cinnamon (125 mg capsule) or placebo: 1.5 gm per day for 6 months	Placebo	↑ homa-ir	Kort and Lobo. (2014a)
	11 patients, age 18–38 years with oligomenorrhea or amenorrhea					
	Placebo group: 6 patients					
	Age 18–38 years					
Polycystic ovarian syndrome	DLBS3233: 18 patients	March 2013 and June 2015 at yasmin clinic, RSCM kencana, jakarta and hasan sadikin hospital, bandung	DLBS3233 (*Cinnamomum burmanii* and *Lagerstroemia speciosa*): 200 mg per day for 6 months metformin group: 1.5 g per day for 6 months	Control	↓AMH level	Wiweko and Susanto. (2017)
	Metformin group: 22 patients					
Polycystic ovarian syndrome	Patients age 23.29 ± 5.10 with rotterdam, overweight or obese	Saudi Arabia	Cinnamon extract (336 mg/day)	Placebo	↓BMI	Talaat and Ammar, (2018)
Polycystic ovary syndrome	40 patients age 18–30 years	2017, ahvaz, Iran	6 weeks	Intervention group	↓weight glucose homeostasis no effects	Parseh et al. (2019)
Polycystic ovary syndrome	Cinnamon group: 20 patients, age 18–42 years with rotterdam criteria	December 2014 to March 2016, national institute of unani medicine (NIUM) hospital, Bengaluru	Cinnamon group: 750 mg capsule,1.5 g/day control group: 500 mg metformin twice a day for 60 days	Control	Menstrual cycle inprovment increased 51.9%, insulin resistance unchanged	Khan and Begum. (2019)

### Neurodegenerative Disorders

Neurodegenerative disorders (ND) include disorders like Alzheimer’s disease, amyotrophic lateral sclerosis, Parkinson's disease, Huntington’s disease, motor neuron disorder, and frontotemporal dementia that result from slow progressive and unalterable drop of certain areas of the nervous system, leading to disruption in nervous system working or death (Nussbaum et al., 2017).

Various *Cinnamomum* species displayed efficacy in the management of neurodegenerative diseases. Several *in vitro* studies are present for cinnamon that can regulate factors that trigger neurodegenerative diseases. Cinnamon extract inhibited Tau aggregation *in vitro* attenuating Alzheimer’s diseases (Peterson et al., 2009). *C. cassia* bark extracted in ethanol demonstrated *in vitro* efficacy against Huntington disease Kaur and Shri (2018). Therefore, cinnamon is necessary to put into clinical trials that develop drugs for neurodegenerative disorders. One clinical trial (NCT03225144) for patients with motor neuron disorder, frontotemporal dementia, or related adult-onset neurodegenerative disorder is ongoing involving 200 participants (Table 3).

### Polycystic Ovary Syndrome: A Possible Control on Its Metabolic Parameters

Polycystic ovary syndrome (PCOS) is caused by an associated endocrine dysfunction and is associated with increased risk of developing insulin resistance, type 2 diabetes, high blood pressure, hypercholesteromy and heart disease.

Several clinical trials reported conflicts in results for cinnamon efficiency on the improvement of BMI, body weight, oxidative stress, and fertility (Hajimonfarednejad et al., 2018a; Borzoei et al., 2018b; Khan and Begum, 2019). Consumption of daily 1.5 g cinnamon did not show any significant effect on BMI and body weight but improved glucose balance in patients with PCOS (Hajimonfarednejad et al., 2018a). However, Borzoei et al. decreased BMI and improved glucose balance treated with the same concentration as applied by Hajimonfarednejad et al. (Hajimonfarednejad et al., 2018a; Borzoei et al., 2018b). Oral supplementation of cinnamon also showed weight loss. This study further exhibited decreased BMI in comparison to the placebo group in PCOS patients (Talaat and Ammar, 2018; Parseh et al., 2019). In contrast to the report by Hajimonfarednejad et al. and Kort and Lobo observed that oral cinnamon consumption did not show any effect on serum glucose balance in PCOS patients (Kort and Lobo, 2014a; Hajimonfarednejad et al., 2018a). This study also supported by Parseh et al., who also did not report any changes in glucose homeostasis (Parseh et al., 2019). All of the studies discussed here showed an effective dose of cinnamon as 1.5 g per day, three times after meal for 1.5–6 months except Talaat and Ammar reported effective dose of 336 mg cinnamon extract per day whereas Wiweko and Susanto (2017) treated with 200 mg of *Cinnamomum burmanii* and *Lagerstroemia speciosa* combination extract (Talaat and Ammar, 2018).

Serum LDL-C, TG and TC levels were also found to be improved in PCOS patients in comparison to placebo (Hajimonfarednejad et al., 2018a; Borzoei et al., 2018b). However, HDL-C level did not show any significant improvement (Borzoei et al., 2018b). Khan and Begum observed no changes in insulin resistance and did not found any significant improvement in patients’ life in comparison with metformin treatment as a control (Khan and Begum, 2019).

Herbal medicine combination extract of *Glycyrrhiza glabra, Paeonia lactiflora, Cinnamomum verum* and *Hypericum perforatum* (supplemented with 1.5 g tablet per day for 3 months) with lifestyle intervention positively improved menstrual regulation and reduction in oligomenorrhoea of 32.9% in patients in comparison with only lifestyle intervention in overweight women with PCOS (Arentz et al., 2017).

Oxidative stress is one of the main causes for increasing lipogenes, BMI in PCOS due to molecular damage, and reduction of serum antioxidants. Borzoei et al. demonstrated that cinnamon extract has antioxidant activity that can improve oxidative stress in women with PCOS (Borzoei et al., 2018a). Table 3 summarized the clinical studies on cinnamon plants in relation to PCOS.

## Toxicological Safety and Adverse Effects of Natural Derivatives of *Cinnamomum* Species

Besides the numerous health benefits, all phyto-therapeutics are not always safe and might result in adverse effects such as allergic dermatitis, the toxicity of organs and interactions with foods and pharmaceuticals (Calixto, 2000). The usual dose for dietary supplements has been suggested to be between 1 and 4 g per day (NIHU, 2015). The usual doses for the administration of cinnamon oil, which is stronger, vary between 50 and 200 mg per day. For doses up to 6 g per day, no adverse reactions were reported (Yun et al., 2018). The common use of cinnamon in food as a spice, food additives and flavoring agent would suggest that it is likely to be safe (Gowder, 2014). However, when consumed in excess, cinnamon can cause respiratory distress, increase pulse rate and increase the sweating process, followed by depressive and drowsy states. This may aggravate the symptoms of rosacea and may increase the risk of developing oral cancer. Coumarin, naturally found in cinnamon, can have a negative influence on the liver, so people with liver disorders should avoid excessive consumption (Organization, 2001).

Cinnamon extract at different doses didn’t produce any toxicity or mortality on rats, as well as no adverse effect was observed (Ahmad et al., 2013) though *trans*-cinnamaldehyde and coumarins are toxic components of cinnamon (Woehrlin et al., 2010; Zhang et al., 2010). High levels of coumarin and cinnamaldehyde might be correlated to liver damage (Deng, 2012), risk of cancer (Abraham et al., 2010), mouth sores (Vivas and Migliari, 2015), low blood sugar (Adisakwattana et al., 2011; Deng, 2012) breathing problems and interaction with certain medications (Abraham et al., 2010). Therefore, the long-term use of a high amount of cinnamon should be continuously monitored. The tolerable daily intake for coumarin (0.1 mg/b. w.) is to be regarded as safe in terms of daily cinnamon intake without the risk of adverse effects (Abraham et al., 2010). When used in large quantities *Cinnamomum* may interact with certain drugs, which could damage the liver.

Used externally, the cinnamon essential oil can produce skin redness, irritation and burning sensation in the *epidermis* (Markman, 2002). In addition, negative interactions with the drugs such as statins, acetaminophen, amiodarone (used to treat heart conditions), carbamazepine (given in the treatment of seizures), medicines for treating fungal infections, methotrexate (used in antitumor treatments) and methyldopa (used in hypertension) may also occur.

Because cinnamon can reduce the concentration of blood sugar, consumed in large quantities, it can interact with diabetes medicines, leading to a very low level of sugar. It is also possible to interfere negatively with tetracycline. Noteworthy, the safety of cinnamon is dependent on supplier quality assurance (SQA), good manufacturing practice (GMP) and good agricultural practice (GAP). It is necessary to control the cultivation, harvesting, plant identification, contamination, adulteration, preparation, packaging, transportation, storage etc. In order to guarantee the safety of the cinnamon products, it is required to assess the quality control of identity/authenticity and purity, stability and shelf life, toxicity physical/chemical/biological/microbial properties and finally standardization of the plant material as well as the products (Kumari and Kotecha, 2016).

## Discussion

One of the strengths of this updated review is that the pharmacological effects highlighted in preclinical *in vitro* studies and in vitvo models could be translated into recent clinical trials in human subjects at well-defined doses, providing strong scientific evidence-based support for therapeutic effects. ethnopharmacological features of cinamomum species.

In addition, current toxicological data show that unwanted side effects may occur at higher doses of cinnamon than shown in pharmacological studies.

Another strength of this comprehensive review is the summarized presentation of the pharmacological mechanisms and molecular targets of action resulting from the meta-analyzes included in the study in order to open new clinical pharmacotherapeutic perspectives.

Starting from traditional uses, pharmacognostic research on *Cinnamomum* plants have identified chemical compounds with biological activities. Three major components found in cinnamon oil and powder: are cinnamaldehyde, acetate, and cinnamyl alcohol, give it beneficial properties. Also, cinnamon is a good source of iron, calcium, manganese and dietary fiber (Vallverdú-Queralt et al., 2014).

Plant organic extracts are a rich source of antimicrobial agents and methanol extracts are known for their antimicrobial properties. Indeed, organic solvents have a good ability to solubilize the active components (de Boer et al., 2005) and their use does not influence the bioactivity of these components (Goyal et al., 2009). (Thongson et al., 2004) also suggested that organic solvents are better solvents of antimicrobial substances. Therefore, a number of preclinical experimental *s*tudies showed the antibacterial effects of *Cinnamomum* plants. Secondary metabolites of Cinnamon plants have the ability to adhere to the microbial cell surface by crossing the lipid layer of the cytoplasmic wall, thus accumulating in the bacterial cell wall. Therefore, phytochemicals alter the structure of the membrane and increase its permeability, causing leakage of vital intracellular metabolites and, finally, cell death (Rhayour, 2003; Wu et al., 2009).

The bacterial cytoplasmic membrane plays a role in selective permeability by ensuring the crossing of H^+^ and K ions^+^. The maintenance and regulation of this barrier are provided by the structural, chemical composition of the cell wall. Increased ion leakage means a disruption of the permeability barrier. Cell metabolism can be influenced by changes in the structural integrity of the cell membrane that, in turn, can lead to the death of cells. (Cox et al., 2001).

Several preclinical experimental studies have been developed to highlight the antidiabetic effects of natural molecules from *Cinnamomum* species. Current conventional therapies used in the treatment of diabetes have many limitations, such as short- and long-term undesirable effects and high rates of failure (Sharifi-Rad et al., 2020a). Therefore, it is necessary to develop more effective drugs to treat diabetes. Presently, complementary herbal remedies are expected to have hypoglycemic properties similar to those of conventional medications without troublesome side effects (Sharifi-Rad et al., 2020b). Traditional herbs and spices can also delay the onset of diabetic complications, control blood sugar levels and correct metabolic abnormalities. The *in vitro* methods are essentially based on the inhibition of enzymes involved in the carbohydrate catabolism and therefore sugar absorption. However, the determination of the *in vivo* antidiabetic activity requires the development of approaches such as oral glucose tolerance test, streptozotocin (SZT)-induced diabetes and alloxan-induced animal models.

The potential mechanisms of antidiabetic effects of Cinnamomum species were highlighted by **r**ecent studies. These have shown that secondary plant metabolites, such as terpenoids and flavonoids, have a significant hypoglycemic effect (Marles and Farnsworth, 1995). Terpenoids stimulate insulin secretion from pancreatic β-cells (Marles and Farnsworth, 1995) whereas cinnamaldehyde has a significantly anti-hyperglycemic effect (Subash Babu et al., 2007).

The antioxidant properties of *Cinnamomum* plants have extensively reported. Polar extracts of the leaves, fruits and seeds, as well as several pure compounds, exhibited relevant activities in a variety of antioxidant assays when compared to positive controls. Cinnamon extracts and its phenolic compounds showed important free radical scavenging properties by the modulation of key enzymes implicated in oxidative stress or by modulation of oxidative pathways that influence the maintaining of redox homeostasis. Cinnamon extracts enhanced ferric reducing antioxidant power (FRAP) and plasma thiol (P-SH), and decreased MDA levels. In addition, they also increased antioxidant enzyme properties such as SOD and catalase (Moselhy and Ali, 2009; Roussel et al., 2009). Our study also showed that Cinnamomum derivatives have a good anti-inflammatory properties.

The antitumor effects of *Cinnamomum* species have been reported by several studies (Koppikar et al., 2010; Kwon et al., 2010; Wondrak et al., 2010; Daker et al., 2013). These antineoplastic activities were tested *in vitro* on several types of human cancer cell lines. These studies highlighted the next possible anticancer mechanisms of *Cinnamomum* plants: 1) inhibition of tumor cells growth (Koppikar et al., 2010), 2) the cinnamon extract is a potent activator of the transcription factor NRF2 (nuclear factor erythroid 2-related factor 2) orchestrated antioxidant response in human cells (Wondrak et al., 2010), 3) cinnamaldehyde involves apoptosis mediated by the increase of p53 and APO-19 (Fas/CD95) protein signaling pathways (Ng and Wu, 2011), 4) DNA (deoxyribonucleic acid) damage (Ng and Wu, 2011), v)apoptosis mediated by the tyrosinase inhibition (Chen et al., 2007; Wang et al., 2011), 6) reducing the mitochondrial transmembrane potential, increasing the ratio of cytochrome C concentration and subsequently activated caspase-9/caspase-3 (Lin et al., 2009).

Recent studies showed neuroprotective effects of *Cinnamomum* plants and their derivatives. The aqueous extract of *C. verum* reduced tau aggregation and filament formation, the markers of Alzheimer’s disease (Peterson et al., 2009). From the brains of patients diagnosed with Alzheimer’s disease, the *C. verum* extract produced the complete disassembly of recombinant tau filaments and caused important alteration of the histology of paired helical filaments isolated, even though it was not deleterious to the normal cellular function of tau. (Peterson et al., 2009). Specifically, the proanthocyanidin, a chemical constituent isolated from the *C. verum* extract was observed to be mainly responsible for this inhibitory activity (Peterson et al., 2009).

Recent clinical studies have found that complementary herbal treatments with cinnamon regulate the frequency of menstrual cycles and may reduce insulin resistance in women with polycystic ovaries.

Regarding the toxicity data, some clinical studies reported that the most common adverse events after cinnamon consumption were gastrointestinal problems in patients with diabetes (Altschuler et al., 2007; Crawford, 2009), *Helicobacter* infection (Nir et al., 2000), polycystic ovarian syndrome (Kort and Lobo, 2014b) and seasonal allergies (Walanj et al., 2014). Dermatitis (Calnan, 1976; Ackermann et al., 2009; Isaac-Renton et al., 2015) and steatites (Miller et al., 1992; Endo and Rees, 2007) were also reported in some case reports. A systematic review of clinical trials and case reports/series on side effects associated with the use of cinnamon in humans indicated that no important difference obtained between cinnamon treated and control group in most cases (Food and Administration, 2005; Hajimonfarednejad et al., 2018b).

Further efforts should be made to investigate future applications of standardized *Cinnamomum* plants using well-designed research.

## Overall Conclusions

*Cinnamomum* plants, its extracts and chemical constituents have several activities promoting human health. Antibacterial, antidiabetic, antioxidant, anti-inflammatory, antitumor and neuroprotective activities are the most studied biological properties of *Cinnamomum* plants. Different from conventional therapeutic medicines, *Cinnamomum* species can be daily consumed in our diet without harmful effects and maybe used as a disease-preventive agent.

In general, chemical constituents of *Cinnamomum* plants such as cinnamon, cinnamaldehyde, cinnamophilin and others, have both direct and indirect activities, *i.e*. antioxidant and antibacterial activities occur by direct action on oxidant species or bacterial, whereas the antidiabetic, anticancer and anti-inflammatory activities occur indirectly via some yet undefined receptor-mediated mechanisms. In this present review, we have retrieved and summarized the wide applications and recent literature on the phytochemistry, bioactivity and pre-clinical and clinical investigations performed on many species of the genus. Besides, a detailed account on their toxicological considerations also indicate their safety and efficacy for human consumption. However, since a plethora of reports exist on the plant species, details on ethnobotany, biotechnological interventions and detailing structure-activity studies are beyond the scope and coverage of the present attempt.

Botanical research advances can improve the obtaining of *Cinnamomum* plants with the best phytochemical compounds. Moreover, the antimicrobial effects of *Cinnamomum* spp. can be potentiated in the food industry as the development of green foods.

## References

[B1] Abdelwahab S. I. Zaman F. Q. Mariod A. A. Yaacob M. Ahmed Abdelmageed A. H. Khamis S. (2010). Chemical Composition, Antioxidant and Antibacterial Properties of the Essential Oils of Etlingera Elatior and Cinnamomum Pubescens Kochummen. J. Sci. Food Agric. 90, 2682–2688. 10.1002/jsfa.4140 20945508

[B2] Abraham K. Wöhrlin F. Lindtner O. Heinemeyer G. Lampen A. (2010). Toxicology and Risk Assessment of Coumarin: Focus on Human Data. Mol. Nutr. Food Res. 54, 228–239. 10.1002/mnfr.200900281 20024932

[B3] Ackermann L. Aalto-Korte K. Jolanki R. Alanko K. (2009). Occupational Allergic Contact Dermatitis from Cinnamon Including One Case from Airborne Exposure. Contact Dermatitis 60, 96–99. 10.1111/j.1600-0536.2008.01486.x 19207380

[B4] Adisakwattana S. Lerdsuwankij O. Poputtachai U. Minipun A. Suparpprom C. (2011). Inhibitory Activity of Cinnamon Bark Species and Their Combination Effect with Acarbose against Intestinal α-glucosidase and Pancreatic α-amylase. Plant Foods Hum. Nutr. 66, 143–148. 10.1007/s11130-011-0226-4 21538147

[B5] Agarwal H. Nakara A. Menon S. Shanmugam V. (2019). Eco-friendly Synthesis of Zinc Oxide Nanoparticles Using Cinnamomum Tamala Leaf Extract and its Promising Effect towards the Antibacterial Activity. J. Drug Deliv. Sci. Tech. 53, 101212. 10.1016/j.jddst.2019.101212

[B6] Ahmad M. Lim C. P. Akowuah G. A. Ismail N. N. Hashim M. A. Hor S. Y. (2013). Safety Assessment of Standardised Methanol Extract of Cinnamomum Burmannii. Phytomedicine 20, 1124–1130. 10.1016/j.phymed.2013.05.005 23827665

[B7] Akilen R. Tsiami A. Devendra D. Robinson N. (2010). Glycated Haemoglobin and Blood Pressure-Lowering Effect of Cinnamon in Multi-Ethnic Type 2 Diabetic Patients in the UK: a Randomized, Placebo-Controlled, Double-Blind Clinical Trial. Diabetic Med. 27, 1159–1167. 10.1111/j.1464-5491.2010.03079.x 20854384

[B8] Al-Bayati F. A. Mohammed M. J. (2009). Isolation, Identification, and Purification of Cinnamaldehyde fromCinnamomum Zeylanicumbark Oil. An Antibacterial Study. Pharm. Biol. 47, 61–66. 10.1080/13880200802430607

[B9] Al-Logmanii A. S. Zari T. A. (2009). Effects of Nigella Sativa L. And Cinnamomum Zeylanicum Blume Oils on Some Physiological Parameters in Streptozotocin-Induced Diabetic Rats. Boletínl Atino Americano y Del. Caribe de Plantas Medicinales y Aromáticas 8, 159.

[B10] Alqasoumi S. (2012). Anti-secretagogue and Antiulcer Effects of Cinnamon Cinnamomum Zeylanicum in Rats. J. Pharmacognosy Phytother. 4, 53–61. 10.5897/jpp12.023

[B11] Altschuler J. A. Casella S. J. Mackenzie T. A. Curtis K. M. (2007). The Effect of Cinnamon on A1C Among Adolescents with Type 1 Diabetes. Diabetes care 30, 813–816. 10.2337/dc06-1871 17392542

[B12] Anand P. Murali K. Y. Tandon V. Murthy P. S. Chandra R. (2010). Insulinotropic Effect of Cinnamaldehyde on Transcriptional Regulation of Pyruvate Kinase, Phosphoenolpyruvate Carboxykinase, and GLUT4 Translocation in Experimental Diabetic Rats. Chemico-Biological Interactions 186, 72–81. 10.1016/j.cbi.2010.03.044 20363216

[B13] Anderson D. Cordell H. J. Fakiola M. Francis R. W. Syn G. Scaman E. S. H. (2015). First Genome-wide Association Study in an Australian Aboriginal Population Provides Insights into Genetic Risk Factors for Body Mass Index and Type 2 Diabetes. PloS one 10, e0119333. 10.1371/journal.pone.0119333 25760438 PMC4356593

[B14] Arentz S. Smith C. A. Abbott J. Fahey P. Cheema B. S. Bensoussan A. (2017). Combined Lifestyle and Herbal Medicine in Overweight Women with Polycystic Ovary Syndrome (PCOS): A Randomized Controlled Trial. Phytother. Res. 31, 1330–1340. 10.1002/ptr.5858 28685911 PMC5599989

[B15] Atta A. H. Alkofahi A. (1998). Anti-nociceptive and Anti-inflammatory Effects of Some Jordanian Medicinal Plant Extracts. J. Ethnopharmacology 60, 117–124. 10.1016/s0378-8741(97)00137-2 9582001

[B16] Azimi P. Ghiasvand R. Feizi A. Hosseinzadeh J. Bahreynian M. Hariri M. (2016). Effect of Cinnamon, Cardamom, Saffron and Ginger Consumption on Blood Pressure and a Marker of Endothelial Function in Patients with Type 2 Diabetes Mellitus: A Randomized Controlled Clinical Trial. Blood Press. 25, 133–140. 10.3109/08037051.2015.1111020 26758574

[B17] Bakar A. Yao P. C. Ningrum V. Liu C. T. Lee S. C. (2020). Beneficial Biological Activities of Cinnamomum Osmophloeum and its Potential Use in the Alleviation of Oral Mucositis: A Systematic Review. Biomedicines 8. 10.3390/biomedicines8010003 PMC7168221 31906292

[B18] Bandara T. Uluwaduge I. Jansz E. R. (2012). Bioactivity of Cinnamon with Special Emphasis on Diabetes Mellitus: a Review. Int. J. Food Sci. Nutr. 63, 380–386. 10.3109/09637486.2011.627849 22007625

[B19] Barceloux D. G. (2009). Cinnamon (Cinnamomum Species). Disease-a-Month 55, 327–335. 10.1016/j.disamonth.2009.03.003 19446676

[B20] Basu S. Jana S. Patel V. B. Patel H. (2013). Effects of Piperine, Cinnamic Acid and Gallic Acid on Rosuvastatin Pharmacokinetics in Rats. Phytother Res. 27, 1548–1556. 10.1002/ptr.4894 23208983

[B21] Bedigian D. (2005). Cinnamon and *Cassia*. The Genus Cinnamomum. Medicinal and Aromatic Plants-Industrial Profiles, Vol. 36. Econ. Bot. 3659, 93–94. 10.1663/0013-0001(2005)059

[B22] Blevins S. M. Leyva M. J. Brown J. Wright J. Scofield R. H. Aston C. E. (2007). Effect of Cinnamon on Glucose and Lipid Levels in Non Insulin-dependent Type 2 Diabetes. Diabetes care 30, 2236–2237. 10.2337/dc07-0098 17563345

[B23] Borges A. Ferreira C. Saavedra M. J. Simões M. (2013). Antibacterial Activity and Mode of Action of Ferulic and Gallic Acids against Pathogenic Bacteria. Microb. Drug Resist. 19, 256–265. 10.1089/mdr.2012.0244 23480526

[B24] Borzoei A. Rafraf M. Asghari-Jafarabadi M. (2018a). Cinnamon Improves Metabolic Factors without Detectable Effects on Adiponectin in Women with Polycystic Ovary Syndrome. Asia Pac. J. Clin. Nutr. 27, 556–563. 10.6133/apjcn.062017.13 29737802

[B25] Borzoei A. Rafraf M. Niromanesh S. Farzadi L. Narimani F. Doostan F. (2018b). Effects of Cinnamon Supplementation on Antioxidant Status and Serum Lipids in Women with Polycystic Ovary Syndrome. J. traditional Complement. Med. 8, 128–133. 10.1016/j.jtcme.2017.04.008 PMC5755995 29322000

[B26] Bösenberg L. H. Van Zyl D. G. (2008). The Mechanism of Action of Oral Antidiabetic Drugs: A Review of Recent Literature. J. Endocrinol. Metab. Diabetes South Africa 13, 80–88. 10.1080/22201009.2008.10872177

[B27] Buga A.-M. Docea A. O. Albu C. Malin R. D. Branisteanu D. E. Ianosi G. (2019). Molecular and Cellular Stratagem of Brain Metastases Associated with Melanoma. Oncol. Lett. 17, 4170–4175. 10.3892/ol.2019.9933 30944612 PMC6444343

[B28] Buru A. S. Pichika M. R. Neela V. Mohandas K. (2014). *In vitro* antibacterial Effects of Cinnamomum Extracts on Common Bacteria Found in Wound Infections with Emphasis on Methicillin-Resistant *Staphylococcus aureus* . J. Ethnopharmacology 153, 587–595. 10.1016/j.jep.2014.02.044 24613273

[B29] Călina D. Docea A. O. Rosu L. Zlatian O. Rosu A. F. Anghelina F. (2017). Antimicrobial Resistance Development Following Surgical Site Infections. Mol. Med. Rep. 15, 681–688. 10.3892/mmr.2016.6034 27959419 PMC5364857

[B30] Calixto J. B. (2000). Efficacy, Safety, Quality Control, Marketing and Regulatory Guidelines for Herbal Medicines (Phytotherapeutic Agents). Braz. J. Med. Biol. Res. 33, 179–189. 10.1590/s0100-879x2000000200004 10657057

[B31] Calnan C. D. (1976). Cinnamon Dermatitis from an Ointment. Contact dermatitis 2, 167–170. 10.1111/j.1600-0536.1976.tb03018.x 139272

[B32] Carson C. F. Mee B. J. Riley T. V. (2002). Mechanism of Action of Melaleuca Alternifolia (Tea Tree) Oil on *Staphylococcus aureus* Determined by Time-Kill, Lysis, Leakage, and Salt Tolerance Assays and Electron Microscopy. Aac 46, 1914–1920. 10.1128/aac.46.6.1914-1920.2002 PMC127210 12019108

[B33] Chakraborty A. Sankaran V. Sankaran M. R. Chellappan D. R. (2015). Chemical Analysis of Leaf Essential Oil of Cinnamomum Verum from Palni Hills, Tamil Nadu. J. Chem. Pharm. Sci. 8, 476–479.

[B34] Chang C.-W. Chang W.-L. Chang S.-T. Cheng S.-S. (2008). Antibacterial Activities of Plant Essential Oils against *Legionella pneumophila* . Water Res. 42, 278–286. 10.1016/j.watres.2007.07.008 17659763

[B35] Chang S.-T. Chen P.-F. Chang S.-C. (2001). Antibacterial Activity of Leaf Essential Oils and Their Constituents from Cinnamomum Osmophloeum. J. Ethnopharmacology 77, 123–127. 10.1016/s0378-8741(01)00273-2 11483389

[B236] Chao S. C. Young D. G. Oberg C. (2000). Screening for Inhibitory Activity of Essential Oils on Selected Bacteria, Fungi and Viruses. J. Essential Oil Res. 12 (5), 639–649. 10.1080/10412905.2000.9712177

[B36] Chen C.-Y. Chen C.-H. Lo Y.-C. Wu B.-N. Wang H.-M. Lo W.-L. (2008). Anticancer Activity of Isoobtusilactone A fromCinnamomum Kotoense: Involvement of Apoptosis, Cell-Cycle Dysregulation, Mitochondria Regulation, and Reactive Oxygen Species. J. Nat. Prod. 71, 933–940. 10.1021/np070620e 18489163

[B37] Chen C.-Y. Chen C.-H. Wong C.-H. Liu Y.-W. Lin Y.-S. Wang Y.-D. (2007). Cytotoxic Constituents of the Stems of Cinnamomum Subavenium. J. Nat. Prod. 70, 103–106. 10.1021/np060425k 17253858

[B38] Chen G. Lu F. Xu L. Dong H. Yi P. Wang F. (2013). The Anti-diabetic Effects and Pharmacokinetic Profiles of Berberine in Mice Treated with Jiao-Tai-Wan and its Compatibility. Phytomedicine 20, 780–786. 10.1016/j.phymed.2013.03.004 23582408

[B39] Chen L. Sun P. Wang T. Chen K. Jia Q. Wang H. (2012). Diverse Mechanisms of Antidiabetic Effects of the Different Procyanidin Oligomer Types of Two Different Cinnamon Species ondb/dbMice. J. Agric. Food Chem. 60, 9144–9150. 10.1021/jf3024535 22920511

[B40] Chen Y. Ma Y. Ma W. (2009). Pharmacokinetics and Bioavailability of Cinnamic Acid after Oral Administration of Ramulus Cinnamomi in Rats. Eur. J. Drug Metabol. Pharmacokinet. 34, 51–56. 10.1007/bf03191384 19462929

[B41] Cheng D. M. Kuhn P. Poulev A. Rojo L. E. Lila M. A. Raskin I. (2012). *In vivo* and In Vitro Antidiabetic Effects of Aqueous Cinnamon Extract and Cinnamon Polyphenol-Enhanced Food Matrix. Food Chem. 135, 2994–3002. 10.1016/j.foodchem.2012.06.117 22980902 PMC3444749

[B42] Chua M.-T. Tung Y.-T. Chang S.-T. (2008). Antioxidant Activities of Ethanolic Extracts from the Twigs of Cinnamomum Osmophloeum. Bioresour. Tech. 99, 1918–1925. 10.1016/j.biortech.2007.03.020 17478090

[B43] Cox S. Mann C. Markham J. Gustafson J. Warmington J. Wyllie S. (2001). Determining the Antimicrobial Actions of Tea Tree Oil. Molecules 6, 87–91. 10.3390/60100087

[B44] Crawford P. (2009). Effectiveness of Cinnamon for Lowering Hemoglobin A1C in Patients with Type 2 Diabetes: a Randomized, Controlled Trial. J. Am. Board Fam. Med. 22, 507–512. 10.3122/jabfm.2009.05.080093 19734396

[B45] Crews R. Gomada Y. Jamison B. Vattem D. (2016). Molecular Effects of Cinnamon Bioactive Compounds for Neuroprotection in D. Melanogaster. FASEB J. 30, 692.

[B46] Daker M. Lin V. Y. Akowuah G. A. Yam M. F. Ahmad M. (2013). Inhibitory Effects of Cinnamomum Burmannii Blume Stem Bark Extract and Trans-cinnamaldehyde on Nasopharyngeal Carcinoma Cells; Synergism with Cisplatin. Exp. Ther. Med. 5, 1701–1709. 10.3892/etm.2013.1041 23837058 PMC3702710

[B47] De Boer H. J. Kool A. Broberg A. Mziray W. R. Hedberg I. Levenfors J. J. (2005). Anti-fungal and Anti-bacterial Activity of Some Herbal Remedies from Tanzania. J. Ethnopharmacology 96, 461–469. 10.1016/j.jep.2004.09.035 15619565

[B48] De Oliveira M. M. M. Brugnera D. F. Do Nascimento J. A. Piccoli R. H. (2012). Control of Planktonic and Sessile Bacterial Cells by Essential Oils. Food Bioproducts Process. 90, 809–818. 10.1016/j.fbp.2012.03.002

[B49] Defronzo R. A. Bonadonna R. C. Ferrannini E. (1992). Pathogenesis of NIDDM: A Balanced Overview. Diabetes Care 15, 318–368. 10.2337/diacare.15.3.318 1532777

[B50] Deng R. (2012). A Review of the Hypoglycemic Effects of Five Commonly Used Herbal Food Supplements. Fna 4, 50–60. 10.2174/1876142911204010050 PMC3626401 22329631

[B51] Di Pasqua R. Betts G. Hoskins N. Edwards M. Ercolini D. Mauriello G. (2007). Membrane Toxicity of Antimicrobial Compounds from Essential Oils. J. Agric. Food Chem. 55, 4863–4870. 10.1021/jf0636465 17497876

[B52] Eidi A. Mortazavi P. Bazargan M. Zaringhalam J. (2012). Hepatoprotective Activity of Cinnamon Ethanolic Extract against CCI4-Induced Liver Injury in Rats. Excli j 11, 495–507. 27547174 PMC4990741

[B53] Endo H. Rees T. D. (2007). Cinnamon Products as a Possible Etiologic Factor in Orofacial Granulomatosis. Med. Oral Patol Oral Cir Bucal 12, E440–E444. 17909510

[B54] Eumkeb G. Siriwong S. Thumanu K. (2012). Synergistic Activity of Luteolin and Amoxicillin Combination against Amoxicillin-Resistant *Escherichia coli* and Mode of Action. J. Photochem. Photobiol. B: Biol. 117, 247–253. 10.1016/j.jphotobiol.2012.10.006 23159507

[B55] EUROPEAN SCIENTIFIC COOPERATIVE ON PHYTOTHERAPY (2003). ESCOP Monographs. 2nd ed. Amsterdam, Netherlands: World Press.

[B56] Fani M. M. Kohanteb J. (2011). Inhibitory Activity of Cinnamon Zeylanicum and *Eucalyptus* Globulus Oils on *Streptococcus* Mutans, *Staphylococcus* Aureus, and *Candida* Species Isolated from Patients with Oral Infections. Shiraz Univ. Dent J. 11, 14–22.

[B57] Farahpour M. Habibi M. (2012). Evaluation of the Wound Healing Activity of an Ethanolic Extract of Ceylon Cinnamon in Mice. Veterinarni Medicina 57, 53–57. 10.17221/4972-vetmed

[B58] Food and Administration 2005. Guidance for Industry: Estimating the Maximum Safe Dose in Initial Clinical Trials for Therapeutics in Adult Healthy Volunteers. Mitochondrion 9. 9–16. 10.1016/j.mito.2008.09.002

[B59] Garcez F. Garcez W. Martins M. Matos M. Guterres Z. Mantovani M. (2005). Cytotoxic and Genotoxic Butanolides and Lignans fromAiouea Trinervis. Planta Med. 71, 923–927. 10.1055/s-2005-871251 16254823

[B60] Geng S. Cui Z. Huang X. Chen Y. Xu D. Xiong P. (2011). Variations in Essential Oil Yield and Composition during Cinnamomum cassia Bark Growth. Ind. Crops Prod. 33, 248–252. 10.1016/j.indcrop.2010.10.018

[B61] Gowder S. (2014). Safety Assessment of Food Flavor-Cinnamaldehyde. Biosafety 3, e147.

[B62] Goyal P. Chauhan A. Kaushik P. (2009). Laboratory Evaluation of Crude Extracts of Cinnamomumtamala for Potential Antibacterial Activity. Electron. J. Biol. 5, 75–79.

[B63] Goyal P. Khanna A. Chauhan A. Chauhan G. Kaushik P. (2008). *In vitro* evaluation of Crude Extracts of Catharanthus Roseus for Potential Antibacterial Activity. Int. J. Green. Pharm. 2, 176–181. 10.4103/0973-8258.42739

[B64] Guerra F. Q. S. Mendes J. M. Sousa J. P. d. Morais-Braga M. F. B. Santos B. H. C. Melo Coutinho H. D. (2012). Increasing Antibiotic Activity against a Multidrug-resistantAcinetobacterspp by Essential Oils ofCitrus limonandCinnamomum Zeylanicum. Nat. Product. Res. 26, 2235–2238. 10.1080/14786419.2011.647019 22191514

[B65] Gutierrez J. L. Bowden R. G. Willoughby D. S. (2016). CassiaCinnamon Supplementation Reduces Peak Blood Glucose Responses but Does Not Improve Insulin Resistance and Sensitivity in Young, Sedentary, Obese Women. J. Dietary Supplements 13, 461–471. 10.3109/19390211.2015.1110222 26716656

[B66] Hajimonfarednejad M. Nimrouzi M. Heydari M. Zarshenas M. M. Raee M. J. Jahromi B. N. (2018a). Insulin Resistance Improvement by Cinnamon Powder in Polycystic Ovary Syndrome: A Randomized Double-Blind Placebo Controlled Clinical Trial. Phytotherapy Res. 32, 276–283. 10.1002/ptr.5970 29250843

[B67] Hajimonfarednejad M. Ostovar M. Raee M. J. Hashempur M. H. Mayer J. G. Heydari M. (2018b). Cinnamon: A Systematic Review of Adverse Events. Clin. Nutr. 38. 594–602. 10.1016/j.clnu.2018.03.013 29661513

[B68] Hameed I. Altameme H. Mohammed G. (2016). Evaluation of Antifungal and Antibacterial Activity and Analysis of Bioactive Phytochemical Compounds of Cinnamomum Zeylanicum (Cinnamon Bark) Using Gas Chromatography-Mass Spectrometry. Orient. J. Chem. 32, 1769–1788. 10.13005/ojc/320406

[B69] Hanhineva K. Törrönen R. Bondia-Pons I. Pekkinen J. Kolehmainen M. Mykkänen H. (2010). Impact of Dietary Polyphenols on Carbohydrate Metabolism. Ijms 11, 1365–1402. 10.3390/ijms11041365 20480025 PMC2871121

[B70] Helander I. M. Alakomi H.-L. Latva-Kala K. Mattila-Sandholm T. Pol I. Smid E. J. (1998). Characterization of the Action of Selected Essential Oil Components on Gram-Negative Bacteria. J. Agric. Food Chem. 46, 3590–3595. 10.1021/jf980154m

[B71] Ho S.-C. Chang K.-S. Chang P.-W. (2013). Inhibition of Neuroinflammation by Cinnamon and its Main Components. Food Chem. 138, 2275–2282. 10.1016/j.foodchem.2012.12.020 23497886

[B72] Hu F. B. (2011). Globalization of Diabetes: the Role of Diet, Lifestyle, and Genes. Diabetes Care 34, 1249–1257. 10.2337/dc11-0442 21617109 PMC3114340

[B73] Hu T. W. Lin Y. T. Ho C. K. (1985). Natural Variation of Chemicalcomponents of the Leaf Oil of Cinnamomum Osmophloeum Kaneh. Bull. Taiwan For. Res. Instustry New Ser. 78, 18.

[B74] Huang D. F. Xu J.-G. Liu J.-X. Zhang H. Hu Q. P. (2014). Chemical Constituents, Antibacterial Activity and Mechanism of Action of the Essential Oil from Cinnamomum cassia Bark against Four Food-Related Bacteria. Microbiology 83, 357–365. 10.1134/s0026261714040067

[B75] Isaac-Renton M. Li M. K. Parsons L. M. (2015). Cinnamon Spice and Everything Not Nice. Dermatitis 26, 116–121. 10.1097/der.0000000000000112 25984687

[B76] Jain S. Sangma T. Shukla S. K. Mediratta P. K. (2015). Effect ofCinnamomum Zeylanicumextract on Scopolamine-Induced Cognitive Impairment and Oxidative Stress in Rats. Nutr. Neurosci. 18, 210–216. 10.1179/1476830514y.0000000113 24559058

[B77] Jang H.-D. Chang K.-S. Huang Y.-S. Hsu C.-L. Lee S.-H. Su M.-S. (2007). Principal Phenolic Phytochemicals and Antioxidant Activities of Three Chinese Medicinal Plants. Food Chem. 103, 749–756. 10.1016/j.foodchem.2006.09.026

[B78] Jantan I. B. Goh S. H. (1992). Essential Oils ofCinnamomumSpecies from Peninsular Malaysia. J. Essent. Oil Res. 4, 161–171. 10.1080/10412905.1992.9698038

[B79] Jantan I. B. Yalvema M. F. Ayop N. Ahmad A. S. (2005). Constituents of the Essential Oils ofCinnamomum Sintoc Blume from a Mountain Forest of Peninsular Malaysia. Flavour Fragr. J. 20, 601–604. 10.1002/ffj.1495

[B80] Jantan I. Ling Y. E. Romli S. Ayop N. Ahmad A. S. (2003). A Comparative Study of the Constituents of the Essential Oils of ThreeCinnamomumSpecies from Malaysia. J. Essent. Oil Res. 15, 387–391. 10.1080/10412905.2003.9698618

[B81] Javed I. Faisal I. Rahman Z. Khan M. Z. Muhammad F. Aslam B. (2012). Lipid Lowering Effect of Cinnamomum Zeylanicum in Hyperlipidaemic Albino Rabbits. Pak J. Pharm. Sci. 25, 141–147. 22186322

[B82] Jayaprakasha G. K. Jagan Mohan Rao L. Sakariah K. K. (2003). Volatile Constituents fromCinnamomum zeylanicumFruit Stalks and Their Antioxidant Activities. J. Agric. Food Chem. 51, 4344–4348. 10.1021/jf034169i 12848508

[B83] Jayaprakasha G. K. Ohnishi-Kameyama M. Ono H. Yoshida M. Jaganmohan Rao L. (2006). Phenolic Constituents in the Fruits ofCinnamomum Zeylanicumand Their Antioxidant Activity. J. Agric. Food Chem. 54, 1672–1679. 10.1021/jf052736r 16506818

[B84] Jia Q. Liu X. Wu X. Wang R. Hu X. Li Y. (2009). Hypoglycemic Activity of a Polyphenolic Oligomer-Rich Extract of Cinnamomum Parthenoxylon Bark in Normal and Streptozotocin-Induced Diabetic Rats. Phytomedicine 16, 744–750. 10.1016/j.phymed.2008.12.012 19464860

[B85] Jiao L. Zhang X. Huang L. Gong H. Cheng B. Sun Y. (2013). Proanthocyanidins Are the Major Anti-diabetic Components of Cinnamon Water Extract. Food Chem. Toxicol. 56, 398–405. 10.1016/j.fct.2013.02.049 23499750

[B86] Kahn S. E. Hull R. L. Utzschneider K. M. (2006). Mechanisms Linking Obesity to Insulin Resistance and Type 2 Diabetes. Nature 444, 840–846. 10.1038/nature05482 17167471

[B87] Kamath J. V. Rana A. C. Roy Chowdhury A. (2003). Pro-healing Effect ofCinnamomum Zeylanicum Bark. Phytother. Res. 17, 970–972. 10.1002/ptr.1293 13680838

[B88] Kamble S. Rambhimaiah S. (2015). Antidiabetic Activity of Aqueous Extract of Cinnamomum cassia in Alloxan-Induced Diabetic Rats. Biomed. Pharmacol. J. 6, 83–88.

[B89] Kang B.-H. Racicot K. Pilkenton S. J. Apostolidis E. (2014). Evaluation of the In Vitro Anti-hyperglycemic Effect of Cinnamomum cassia Derived Phenolic Phytochemicals, via Carbohydrate Hydrolyzing Enzyme Inhibition. Plant Foods Hum. Nutr. 69, 155–160. 10.1007/s11130-014-0415-z 24706251

[B90] Kannappan S. Jayaraman T. Rajasekar P. Ravichandran M. K. Anuradha C. V. (2006). Cinnamon Bark Extract Improves Glucose Metabolism and Lipid Profile in the Fructose-Fed Rat. Singapore Med. J. 47, 858–863. 16990960

[B91] Kar A. Choudhary B. K. Bandyopadhyay N. G. (2003). Comparative Evaluation of Hypoglycaemic Activity of Some Indian Medicinal Plants in Alloxan Diabetic Rats. J. Ethnopharmacology 84, 105–108. 10.1016/s0378-8741(02)00144-7 12499084

[B92] Kaul P. N. Bhattacharya A. K. Rajeswara Rao B. R. Syamasundar K. V. Ramesh S. (2003). Volatile Constituents of Essential Oils Isolated from Different Parts of Cinnamon (Cinnamomum Zeylanicum Blume). J. Sci. Food Agric. 83, 53–55. 10.1002/jsfa.1277

[B237] Kaur R. Shri R. (2018). Role of the Genus Cinnamomum in the Management of Neurodegenerative Diseases: Outcomes and Shortcomings. Indian J. Pharm. Sci. 80 (6), 984–995. 10.4172/pharmaceutical-sciences.1000448

[B93] Khan A. A. Begum W. (2019). Efficacy of Darchini in the Management of Polycystic Ovarian Syndrome: A Randomized Clinical Study. J. Herbal Med. 15, 100249. 10.1016/j.hermed.2018.11.005

[B94] Khan A. Safdar M. Ali Khan M. M. Khattak K. N. Anderson R. A. (2003). Cinnamon Improves Glucose and Lipids of People with Type 2 Diabetes. Diabetes care 26, 3215–3218. 10.2337/diacare.26.12.3215 14633804

[B95] Kim D. H. Kim C. H. Kim M.-S. Kim J. Y. Jung K. J. Chung J. H. (2007). Suppression of Age-Related Inflammatory NF-Κb Activation by Cinnamaldehyde. Biogerontology 8, 545–554. 10.1007/s10522-007-9098-2 17486422

[B96] Kim H.-O. Park S.-W. Park H.-D. (2004). Inactivation of *Escherichia coli* O157:H7 by Cinnamic Aldehyde Purified from Cinnamomum cassia Shoot. Food Microbiol. 21, 105–110. 10.1016/s0740-0020(03)00010-8

[B97] Kim S. H. Hyun S. H. Choung S. Y. (2006). Antioxidative Effects ofCinnamomi cassiaeandRhodiola Roseaextracts in Liver of Diabetic Mice. Biofactors 26, 209–219. 10.1002/biof.5520260306 16971752

[B98] King H. Aubert R. E. Herman W. H. (1998). Global Burden of Diabetes, 1995-2025: Prevalence, Numerical Estimates, and Projections. Diabetes Care 21, 1414–1431. 10.2337/diacare.21.9.1414 9727886

[B99] Koppikar S. J. Choudhari A. S. Suryavanshi S. A. Kumari S. Chattopadhyay S. Kaul-Ghanekar R. (2010). Aqueous Cinnamon Extract (ACE-C) from the Bark of Cinnamomum cassia Causes Apoptosis in Human Cervical Cancer Cell Line (SiHa) through Loss of Mitochondrial Membrane Potential. BMC Cancer 10, 210. 10.1186/1471-2407-10-210 20482751 PMC2893107

[B100] Kort D. H. Lobo R. A. (2014a). Preliminary Evidence that Cinnamon Improves Menstrual Cyclicity in Women with Polycystic Ovary Syndrome: a Randomized Controlled Trial. Am. J. Obstet. Gynecol. 211, 487–496. 10.1016/j.ajog.2014.05.009e1 24813595

[B102] Krishnakumar I. M. Abin I. Johannah N. M. Eapen N. Balu M. Ramadassan K. (2014). Effects of the Polyphenol Content on the Anti-diabetic Activity of Cinnamomum Zeylanicum Extracts. Food Funct. 5, 2208–2220. 25051315 10.1039/c4fo00130c

[B103] Kumar S. Vasudeva N. Sharma S. (2012). GC-MS Analysis and Screening of Antidiabetic, Antioxidant and Hypolipidemic Potential of Cinnamomum Tamala Oil in Streptozotocin Induced Diabetes Mellitus in Rats. Cardiovasc. Diabetol. 11, 95. 10.1186/1475-2840-11-95 22882757 PMC3461457

[B104] Kumari R. Kotecha M. (2016). A Review on the Standardization of Herbal Medicines. Int. J. Pharm. Sci. Res. 7, 97–106. 10.7897/2230-8407.07319

[B105] Kuo S.-Y. Hsieh T.-J. Wang Y.-D. Lo W.-L. Hsui Y.-R. Chen C.-Y. (2008). Cytotoxic Constituents from the Leaves of Cinnamomum Subavenium. Chem. Pharm. Bull. 56, 97–101. 10.1248/cpb.56.97 18175985

[B106] Kwon H.-K. Hwang J.-S. So J.-S. Lee C.-G. Sahoo A. Ryu J.-H. (2010). Cinnamon Extract Induces Tumor Cell Death through Inhibition of NFκB and AP1. BMC cancer 10, 392. 10.1186/1471-2407-10-392 20653974 PMC2920880

[B107] Lambert R. J. W. Skandamis P. N. Coote P. J. Nychas G.-J. E. (2001). A Study of the Minimum Inhibitory Concentration and Mode of Action of Oregano Essential Oil, Thymol and Carvacrol. J. Appl. Microbiol. 91, 453–462. 10.1046/j.1365-2672.2001.01428.x 11556910

[B108] Latta C. H. Brothers H. M. Wilcock D. M. (2015). Neuroinflammation in Alzheimer's Disease; A Source of Heterogeneity and Target for Personalized Therapy. Neuroscience 302, 103–111. 10.1016/j.neuroscience.2014.09.061 25286385 PMC4602369

[B109] Lee H.-S. Ahn Y.-J. (1998). Growth-Inhibiting Effects ofCinnamomum cassiaBark-Derived Materials on Human Intestinal Bacteria. J. Agric. Food Chem. 46, 8–12. 10.1021/jf970548y 10554188

[B110] Lee H. J. Hyun E.-A. Yoon W. J. Kim B. H. Rhee M. H. Kang H. K. (2006). *In vitro* anti-inflammatory and Anti-oxidative Effects of Cinnamomum Camphora Extracts. J. Ethnopharmacology 103, 208–216. 10.1016/j.jep.2005.08.009 16182479

[B111] Lee M.-G. Kuo S.-Y. Yen S.-Y. Hsu H.-F. Leung C.-H. Ma D.-L. (2015). Evaluation of Cinnamomum Osmophloeum Kanehira Extracts on Tyrosinase Suppressor, Wound Repair Promoter, and Antioxidant. Scientific World J. 7. 303415. 10.1155/2015/303415 PMC4370200 25839053

[B112] Leela N. K. (2008). “Cinnamon and cassia,” in Chemistry of Spices. Editors PARTHASARATHY V. A. CHEMPAKAM B. ZACHARIAH T. J. (Wallingford, Oxfordshire, UK: CAB International).

[B113] Leiter L. Lewanczuk R. (2005). Of the Renin-Angiotensin System and Reactive Oxygen speciesType 2 Diabetes and Angiotensin II Inhibition. Am. J. Hypertens. 18, 121–128. 10.1016/j.amjhyper.2004.07.001 15691626

[B114] Li L. Li Z. W. Yin Z. Q. Wei Q. Jia R. Y. Zhou L. J. (2014). Antibacterial Activity of Leaf Essential Oil and its Constituents from Cinnamomum Longepaniculatum. Int. J. Clin. Exp. Med. 7, 1721–1727. 25126170 PMC4132134

[B115] Liao B.-C. Hsieh C.-W. Liu Y.-C. Tzeng T.-T. Sun Y.-W. Wung B.-S. (2008). Cinnamaldehyde Inhibits the Tumor Necrosis Factor-α-Induced Expression of Cell Adhesion Molecules in Endothelial Cells by Suppressing NF-Κb Activation: Effects upon IκB and Nrf2. Toxicol. Appl. Pharmacol. 229, 161–171. 10.1016/j.taap.2008.01.021 18304597

[B116] Liao J.-C. Deng J.-S. Chiu C.-S. Hou W.-C. Huang S.-S. Shie P.-H. (2012a). Anti-inflammatory Activities of Cinnamomum cassia Constituents In Vitro and In Vivo. Evidence-Based Complement. Altern. Med. 2012, 429320. 10.1155/2012/429320 PMC3318905 22536283

[B117] Liao J. C. Deng J. S. Chiu C. S. Hou W. C. Huang S. S. Shie P. H. (2012b). Anti-Inflammatory Activities of Cinnamomum cassia Constituents *In Vitro* and *In Vivo* . Evid. Based Complement. Alternat Med. 2012, 429320. 10.1155/2012/429320 22536283 PMC3318905

[B118] Lin C.-C. Wu S.-J. Chang C.-H. Ng L.-T. (2003). Antioxidant Activity ofCinnamomum cassia. Phytother. Res. 17, 726–730. 10.1002/ptr.1190 12916067

[B119] Lin G.-M. Chen Y.-H. Yen P.-L. Chang S.-T. (2016). Antihyperglycemic and Antioxidant Activities of Twig Extract from Cinnamomum Osmophloeum. J. Traditional Complement. Med. 6, 281–288. 10.1016/j.jtcme.2015.08.005 PMC4936769 27419094

[B120] Lin K. Yeh S. Lin M. Shih M. Yang K. Hwang S. (2007). Major Chemotypes and Antioxidative Activity of the Leaf Essential Oils of Cinnamomum Osmophloeum Kaneh. From a Clonal Orchard. Food Chem. 105, 133–139. 10.1016/j.foodchem.2007.03.051

[B121] Lin R.-J. Cheng M.-J. Huang J.-C. Lo W.-L. Yeh Y.-T. Yen C.-M. (2009). Cytotoxic Compounds from the Stems ofCinnamomum Tenuifolium. J. Nat. Prod. 72, 1816–1824. 10.1021/np900225p 19754130

[B122] Loy G. Cottiglia F. Garau D. Deidda D. Pompei R. Bonsignore L. (2001). Chemical Composition and Cytotoxic and Antimicrobial Activity of Calycotome Villosa (Poiret) Link Leaves. Il Farmaco 56, 433–436. 10.1016/s0014-827x(01)01056-4 11482772

[B123] Lu Z. Jia Q. Wang R. Wu X. Wu Y. Huang C. (2011). Hypoglycemic Activities of A- and B-type Procyanidin Oligomer-Rich Extracts from Different Cinnamon Barks. Phytomedicine 18, 298–302. 10.1016/j.phymed.2010.08.008 20851586

[B124] Luscher T. F. Steffel J. (2008). Sweet and Sour. Circ. Res. 102, 9–11. 10.1161/01.res.0000303937.73170.31 18174471

[B125] Mamindla S. Srgp Koganti V. Ravouru N. Koganti B. (2017). Effect of Cinnamomum cassia on the Pharmacokinetics and Pharmacodynamics of Pioglitazone. Curr. Clin. Pharmacol. 12, 41–49. 10.2174/1574884712666170207152020 28176623

[B126] Mang B. Wolters M. Schmitt B. Kelb K. Lichtinghagen R. Stichtenoth D. O. (2006). Effects of a Cinnamon Extract on Plasma Glucose, HbA1c, and Serum Lipids in Diabetes Mellitus Type 2. Eur. J. Clin. Invest. 36, 340–344. 10.1111/j.1365-2362.2006.01629.x 16634838

[B127] Markman M. (2002). Safety Issues in Using Complementary and Alternative Medicine. J. Clin. Oncol. 20, 39S–41S. 12235223

[B128] Marles R. J. Farnsworth N. R. (1995). Antidiabetic Plants and Their Active Constituents. Phytomedicine 2, 137–189. 10.1016/s0944-7113(11)80059-0 23196156

[B129] Mathew S. Abraham T. E. (2006). Studies on the Antioxidant Activities of Cinnamon (Cinnamomum Verum) Bark Extracts, through Various In Vitro Models. Food Chem. 94, 520–528. 10.1016/j.foodchem.2004.11.043

[B130] Mbwambo Z. H. Moshi M. J. Masimba P. J. Kapingu M. C. Nondo R. S. (2007). Antimicrobial Activity and Brine Shrimp Toxicity of Extracts of *Terminalia* Brownii Roots and Stem. BMC Complement. Altern. Med. 7, 9. 10.1186/1472-6882-7-9 17394672 PMC1851717

[B131] Miller R. L. Gould A. R. Bernstein M. L. (1992). Cinnamon-induced Stomatitis Venenata. Oral Surg. Oral Med. Oral Pathol. 73, 708–716. 10.1016/0030-4220(92)90016-j 1437042

[B132] Mishra A. Bhatti R. Singh A. Singh Ishar M. (2010). Ameliorative Effect of the Cinnamon Oil fromCinnamomum Zeylanicumupon Early Stage Diabetic Nephropathy. Planta Med. 76, 412–417. 10.1055/s-0029-1186237 19876811

[B133] Moselhy S. S. Ali H. K. (2009). Hepatoprotective Effect of Cinnamon Extracts against Carbon Tetrachloride Induced Oxidative Stress and Liver Injury in Rats. Biol. Res. 42, 93–98. 10.4067/s0716-97602009000100009 19621136

[B134] Ng L. T. Wu S. J. (2011). Antiproliferative Activity of Cinnamomum cassia Constituents and Effects of Pifithrin-Alpha on Their Apoptotic Signaling Pathways in Hep G2 Cells. Evid. Based Complement. Alternat Med. 2011, 492148. 10.1093/ecam/nep220 20038571 PMC3135661

[B135] Nihu S. (2015). Department of Health and Human Services Herbs at a Glance. USA: Cinnamon NIH.

[B136] Nir Y. Potasman I. Stermer E. Tabak M. Neeman I. (2000). Controlled Trial of the Effect of Cinnamon Extract on Helicobacter pylori. Helicobacter 5, 94–97. 10.1046/j.1523-5378.2000.00014.x 10849058

[B137] Noudeh G. D. Sharififar F. Noodeh A. D. Moshafi M. H. Afzadi M. A. Behravan E. (2010). Antitumor and Antibacterial Activity of Four Fractions from Heracleumpersicum Desf. And Cinnamomum Zeylanicum Blume. J. Med. Plants Res. 4, 2176–2180.

[B138] Nussbaum L. Hogea L. M. Călina D. Andreescu N. Grădinaru R. S,tefănescu R. (2017). Modern Treatment Approaches in Psychoses. Pharmacogenetic, Neuroimagistic and Clinical Implications. Farmacia 65, 75–81.

[B139] Nyadjeu P. Dongmo A. Nguelefack T. B. Kamanyi A. (2011). Antihypertensive and Vasorelaxant Effects of Cinnamomum Zeylanicum Stem Bark Aqueous Extract in Rats. J. Complement. Integr. Med. 8. 10.2202/1553-3840.1490 22754922

[B140] Ooi L. S. M. Li Y. Kam S.-L. Wang H. Wong E. Y. L. Ooi V. E. C. (2006). Antimicrobial Activities of Cinnamon Oil and Cinnamaldehyde from the Chinese Medicinal HerbCinnamomum cassiaBlume. Am. J. Chin. Med. 34, 511–522. 10.1142/s0192415x06004041 16710900

[B141] ORGANIZATION (2001). Joint FAO/WHO Expert Committee on Food Additives. Safety evaluation of certain food additives and contaminants in food: Fumonisins. Proceedings of the 56th Meeting of the Joint FAO/WHO Expert Committee on Food Additives, 103–279.

[B142] Ortiz-Andrade R. R. García-Jiménez S. Castillo-España P. Ramírez-Ávila G. Villalobos-Molina R. Estrada-Soto S. (2007). α-Glucosidase Inhibitory Activity of the Methanolic Extract from Tournefortia Hartwegiana: An Anti-hyperglycemic Agent. J. Ethnopharmacology 109, 48–53. 10.1016/j.jep.2006.07.002 16920301

[B143] Oussalah M. Caillet S. Saucier L. Lacroix M. (2006). Antimicrobial Effects of Selected Plant Essential Oils on the Growth of a *Pseudomonas* Putida Strain Isolated from Meat. Meat Sci. 73, 236–244. 10.1016/j.meatsci.2005.11.019 22062294

[B144] Padureanu R. Albu C. V. Mititelu R. R. Bacanoiu M. V. Docea A. O. Calina D. (2019). Oxidative Stress and Inflammation Interdependence in Multiple Sclerosis. Jcm 8, 1815. 10.3390/jcm8111815 PMC6912446 31683787

[B145] Panickar K. S. Polansky M. M. Anderson R. A. (2009). Cinnamon Polyphenols Attenuate Cell Swelling and Mitochondrial Dysfunction Following Oxygen-Glucose Deprivation in Glial Cells. Exp. Neurol. 216, 420–427. 10.1016/j.expneurol.2008.12.024 19166834

[B146] Panickar K. S. Polansky M. M. Graves D. J. Urban J. F. JR. Anderson R. A. (2012). A Procyanidin Type A Trimer from Cinnamon Extract Attenuates Glial Cell Swelling and the Reduction in Glutamate Uptake Following Ischemia-like Injury In Vitro. Neuroscience 202, 87–98. 10.1016/j.neuroscience.2011.11.051 22166344

[B147] Parseh S. Shakerian S. Alizadeh A. A. (2019). Effect of Chronic Aerobic/Resistive Exercises with Supplementation of Cinnamon on Insulin Resistance in Women with Polycystic Ovary Syndrome in Ahvaz City in 2017. J. Arak Univ. Med. Sci. 22, 15–26.

[B148] Peng X. Cheng K.-W. Ma J. Chen B. Ho C.-T. Lo C. (2008). Cinnamon Bark Proanthocyanidins as Reactive Carbonyl Scavengers to Prevent the Formation of Advanced Glycation Endproducts. J. Agric. Food Chem. 56, 1907–1911. 10.1021/jf073065v 18284204

[B149] Peterson D. W. George R. C. Scaramozzino F. Lapointe N. E. Anderson R. A. Graves D. J. (2009). Cinnamon Extract Inhibits Tau Aggregation Associated with Alzheimer's Disease In Vitro. Jad 17, 585–597. 10.3233/jad-2009-1083 19433898

[B150] Prasad K. N. Yang B. Dong X. Jiang G. Zhang H. Xie H. (2009). Flavonoid Contents and Antioxidant Activities from Cinnamomum Species. Innovative Food Sci. Emerging Tech. 10, 627–632. 10.1016/j.ifset.2009.05.009

[B151] Pyo J.-H. Jeong Y.-K. Yeo S. Lee J.-H. Jeong M.-Y. Kim S.-H. (2013). Neuroprotective Effect of Trans-cinnamaldehyde on the 6-Hydroxydopamine-Induced Dopaminergic Injury. Biol. Pharm. Bull. 36, 1928–1935. 10.1248/bpb.b13-00537 24292051

[B152] Qin B. Panickar K. S. Anderson R. A. (2014). Cinnamon Polyphenols Regulate S100β, Sirtuins, and Neuroactive Proteins in Rat C6 Glioma Cells. Nutrition 30, 210–217. 10.1016/j.nut.2013.07.001 24239092

[B153] Qin B. Panickar K. S. Anderson R. A. (2010). Cinnamon: Potential Role in the Prevention of Insulin Resistance, Metabolic Syndrome, and Type 2 Diabetes. J. Diabetes Sci. Technol. 4, 685–693. 10.1177/193229681000400324 20513336 PMC2901047

[B154] Radhia K. Zakkia K. Shah S. H. (2010). Cinnamon May Reduce Glucose, Lipid and Cholesterol Level in Type 2 Diabetic Individuals. Pakistan J. Nutr. 9, 430–433.

[B155] Raina V. K. Srivastava S. K. Aggarwal K. K. Ramesh S. Kumar S. (2001). Essential Oil Composition ofCinnamomum Zeylanicum Blume Leaves from Little Andaman, India. Flavour Fragr. J. 16, 374–376. 10.1002/ffj.1016

[B156] Rajbir B. Kaur S. Singh J. ISHARMPS (2009). Ameliorative Effect of Volatile Oil from Cinnamomum Zeylanicum on Hyperalgesia in Alloxan Diabetic Rats. Can. J. Pure Applsci 3, 887–895.

[B157] Rameshkumar K. B. George V. Shiburaj S. (2007). Chemical Constituents and Antibacterial Activity of the Leaf Oil ofCinnamomum chemungianumMohan et Henry. J. Essent. Oil Res. 19, 98–100. 10.1080/10412905.2007.9699238

[B158] Ranasinghe P. Jayawardana R. Galappaththy P. Constantine G. R. De Vas Gunawardana N. Katulanda P. (2012). Efficacy and Safety of 'true' cinnamon(Cinnamomum Zeylanicum)as a Pharmaceutical Agent in Diabetes: a Systematic Review and Meta-Analysis. Diabet Med. 29, 1480–1492. 10.1111/j.1464-5491.2012.03718.x 22671971

[B159] Ranilla L. G. Kwon Y.-I. Apostolidis E. Shetty K. (2010). Phenolic Compounds, Antioxidant Activity and In Vitro Inhibitory Potential against Key Enzymes Relevant for Hyperglycemia and Hypertension of Commonly Used Medicinal Plants, Herbs and Spices in Latin America. Bioresour. Tech. 101, 4676–4689. 10.1016/j.biortech.2010.01.093 20185303

[B160] Ranjbar A. Ghaseminejhad S. Takalu H. Baiaty A. Rahimi F. Abdollahi M. (2007). Anti Oxidative Stress Potential of Cinnamon (Cinnamomum Zeylanicum) in Operating Room Personnel; A Before/After Cross Sectional Clinical Trial. Int. J. Pharmacol. 3, 482–486.

[B161] Rao H. J. LAKSHMI (2012). Anti-diarrhoeal Activity of the Aqueous Extract of the Bark of Cinnamomum Zeylanicum Linn in Mice. J. Clin. Diag Res. 6, 215–219.

[B162] Rao P. V. Gan S. H. (2014). Cinnamon: A Multifaceted Medicinal Plant. Evidence-Based Complement. Altern. Med. 2014, 642942. 10.1155/2014/642942 PMC4003790 24817901

[B163] Rasheed M. U. Thajuddin N. (2011). Effect of Medicinal Plants on Moraxella Cattarhalis. Asian Pac. J. Trop. Med. 4, 133–136. 10.1016/S1995-7645(11)60053-9 21771437

[B164] Rashidi M. Malekirad A. A. Abdollahi M. Habibollahi S. Dolatyari N. Narimani M. (2014). The Effect of Tea-Cinnamon and Melissa Officinalis L. Aqueous Extraction, on Neuropsychology Distress, Biochemical and Oxidative Stress Biomarkers in Glass Production Workers. Health 06, 2592–2601. 10.4236/health.2014.619298

[B165] Ravindran P. Nirmal-Babu K. Shylaja M. (2003). Cinnamon and cassia: The Genus Cinnamomum. CRC Press.

[B166] Rhayour K. Bouchikhi T. Tantaoui-Elaraki A. Sendide K. Remmal A. (2003). The Mechanism of Bactericidal Action of Oregano and Clove Essential Oils and of Their Phenolic Major Components onEscherichia coliandBacillus Subtilis. J. Essent. Oil Res. 15 (4), 286–292. 10.1080/10412905.2003.9712144

[B167] Ribeiro-Santos R. Andrade M. Madella D. Martinazzo A. P. de Aquino Garcia Moura L. De Melo N. R. (2017). Revisiting an Ancient Spice with Medicinal Purposes: Cinnamon. Trends Food Sci. Tech. 62, 154–169. 10.1016/j.tifs.2017.02.011

[B168] Rogoveanu O. C. Calina D. Cucu M. G. Burada F. Docea A. O. Sosoi S. (2018). Association of Cytokine Gene Polymorphisms with Osteoarthritis Susceptibility. Exp. Ther. Med. 16, 2659–2664. 10.3892/etm.2018.6477 30186498 PMC6122495

[B169] Ross M. S. F. (1976). Analysis of Cinnamon Oils by High-Pressure Liquid Chromatography. J. Chromatogr. A 118, 273–275. 10.1016/s0021-9673(00)81222-4

[B170] Rossi C. Chaves-López C. Možina S. S. Di Mattia C. Scuota S. Luzzi I. (2019). Salmonella enterica Adhesion: Effect of Cinnamomum Zeylanicum Essential Oil on Lettuce. LWT 111, 16–22. 10.1016/j.lwt.2019.05.026

[B171] Roussel A.-M. Hininger I. Benaraba R. Ziegenfuss T. N. Anderson R. A. (2009). Antioxidant Effects of a Cinnamon Extract in People with Impaired Fasting Glucose that Are Overweight or Obese. J. Am. Coll. Nutr. 28, 16–21. 10.1080/07315724.2009.10719756 19571155

[B172] Russell A. D. (2002). Antibiotic and Biocide Resistance in Bacteria: Introduction. J. Appl. Microbiol. 92 (Suppl. l), 1s–3s. 10.1046/j.1365-2672.92.5s1.14.x 12000607

[B173] Sadeghi S. Davoodvandi A. Pourhanifeh M. H. Sharifi N. Arefnezhad R. Sahebnasagh R. (2019). Anti-cancer Effects of Cinnamon: Insights into its Apoptosis Effects. Eur. J. Med. Chem. 178, 131–140. 10.1016/j.ejmech.2019.05.067 31195168

[B174] Salehi B. Capanoglu E. Adrar N. Catalkaya G. Shaheen S. Jaffer M. (2019a). Cucurbits Plants: A Key Emphasis to its Pharmacological Potential. Molecules 24, 1854. 10.3390/molecules24101854 PMC6572650 31091784

[B175] Salehi B. Jornet P. L. Lopez E. P. F. Calina D. Sharifi-Rad M. Ramirez-Alarcon K. (2019b). Plant-Derived Bioactives in Oral Mucosal Lesions: A Key Emphasis to Curcumin, Lycopene, Chamomile, Aloe Vera, Green Tea and Coffee Properties. Biomolecules 9, 23. 10.3390/biom9030106 PMC6468600 30884918

[B176] Salehi B. Rescigno A. Dettori T. Calina D. Docea A. O. Singh L. (2020a). Avocado-Soybean Unsaponifiables: A Panoply of Potentialities to Be Exploited. Biomolecules 10, 130. 10.3390/biom10010130 PMC7023362 31940989

[B177] Salehi B. Sharifi-Rad J. Capanoglu E. Adrar N. Catalkaya G. Shaheen S. (2019c). Cucurbita Plants: From Farm to Industry. Appl. Sci. 9, 3387. 10.3390/app9163387

[B178] Salehi B. Sharifi-Rad J. Cappellini F. Reiner A. Zorzan D. Imran M. (2020b). The Therapeutic Potential of Anthocyanins: Current Approaches Based on Their Molecular Mechanism of Action. Front. Pharmacol. 11, 20. 10.3389/fphar.2020.01300 32982731 PMC7479177

[B179] Salehi B. Shivaprasad Shetty M. V. Anil Kumar N. Živković J. Calina D. Oana Docea A. (2019d). Veronica Plants-Drifting from Farm to Traditional Healing, Food Application, and Phytopharmacology. Molecules 24, 2454. 10.3390/molecules24132454 PMC6651156 31277407

[B180] Samarasekera R. Kalhari K. S. Weerasinghe I. S. (2005). Mosquitocidal Activity of Leaf and Bark Essential Oils of CeylonCinnamomum Zeylanicum. J. Essent. Oil Res. 17, 301–303. 10.1080/10412905.2005.9698909

[B181] Senanayake U. M. Lee T. H. Wills R. B. H. (1978). Volatile Constituents of Cinnamon (Cinnamomum Zeylanicum) Oils. J. Agric. Food Chem. 26, 822–824. 10.1021/jf60218a031

[B182] Shan B. Cai Y.-Z. Brooks J. D. Corke H. (2007). Antibacterial Properties and Major Bioactive Components of Cinnamon Stick (Cinnamomum Burmannii): Activity against Foodborne Pathogenic Bacteria. J. Agric. Food Chem. 55, 5484–5490. 10.1021/jf070424d 17567030

[B183] Sharifi-Rad J. Rodrigues C. F. Sharopov F. Docea A. O. Karaca A. C. Sharifi-Rad M. (2020a). Diet, Lifestyle and Cardiovascular Diseases: Linking Pathophysiology to Cardioprotective Effects of Natural Bioactive Compounds. Int. J. Environ. Res. Public Health 17, 31. 10.3390/ijerph17072326 PMC7177934 32235611

[B184] Sharifi-Rad M. Kumar N. V. A. Zucca P. Varoni E. M. Dini L. Panzarini E. (2020b). Lifestyle, Oxidative Stress, and Antioxidants: Back and Forth in the Pathophysiology of Chronic Diseases. Front. Physiol. 11, 21. 10.3389/fphys.2020.00694 32714204 PMC7347016

[B185] Shen Y. Fukushima M. Ito Y. Muraki E. Hosono T. Seki T. (2010). Verification of the Antidiabetic Effects of Cinnamon (Cinnamomum Zeylanicum) Using Insulin-Uncontrolled Type 1 Diabetic Rats and Cultured Adipocytes. Biosci. Biotechnol. Biochem. 74, 2418–2425. 10.1271/bbb.100453 21150113

[B186] Shihabudeen H. M. S. Priscilla D. H. Thirumurugan K. (2011). Cinnamon Extract Inhibits Alpha-Glucosidase Activity and Dampens Postprandial Glucose Excursion in Diabetic Rats. Nutr. Metab. (Lond) 8, 46. 21711570 10.1186/1743-7075-8-46 PMC3155477

[B187] Shishehbor F. Rezaeyan Safar M. Rajaei E. Haghighizadeh M. H. (2018). Cinnamon Consumption Improves Clinical Symptoms and Inflammatory Markers in Women with Rheumatoid Arthritis. J. Am. Coll. Nutr. 37, 685–690. 10.1080/07315724.2018.1460733 29722610

[B188] Singh G. Maurya S. Delampasona M. P. Catalan C. A. N. (2007). A Comparison of Chemical, Antioxidant and Antimicrobial Studies of Cinnamon Leaf and Bark Volatile Oils, Oleoresins and Their Constituents. Food Chem. Toxicol. 45, 1650–1661. 10.1016/j.fct.2007.02.031 17408833

[B189] Skandamis P. N. Nychas G.-J. E. (2001). Effect of Oregano Essential Oil on Microbiological and Physico-Chemical Attributes of Minced Meat Stored in Air and Modified Atmospheres. J. Appl. Microbiol. 91, 1011–1022. 10.1046/j.1365-2672.2001.01467.x 11851808

[B190] Stalnikowitz D. K. Weissbrod A. B. (2003). Liver Fibrosis and Inflammation. A Review. Ann. Hepatol. 2, 159–163. 10.1016/s1665-2681(19)32127-1 15115954

[B191] Subash Babu P. Prabuseenivasan S. Ignacimuthu S. (2007). Cinnamaldehyde-A Potential Antidiabetic Agent. Phytomedicine 14, 15–22. 10.1016/j.phymed.2006.11.005 17140783

[B192] Suppapitiporn S. Kanpaksi N. (2006). The Effect of Cinnamon cassia Powder in Type 2 Diabetes Mellitus. J. Med. Assoc. Thai 89 Suppl 3, S200–S205. 17718288

[B193] Takasao N. Tsuji-Naito K. Ishikura S. Tamura A. Akagawa M. (2012). Cinnamon Extract Promotes Type I Collagen Biosynthesis via Activation of IGF-I Signaling in Human Dermal Fibroblasts. J. Agric. Food Chem. 60, 1193–1200. 10.1021/jf2043357 22233457

[B194] Talaat B. Ammar I. M. M. (2018). The Added Value of Cinnamon to Metformin in Controlling Symptoms of Polycystic Ovary Syndrome, a Randomized Controlled Trial. Middle East Fertil. Soc. J. 23, 440–445. 10.1016/j.mefs.2018.03.005

[B195] Tao Y. Xu X. Yan J. Cai B. (2019). A Sensitive UPLC–MS/MS Method for Simultaneous Determination of Polyphenols in Rat Plasma: Application to a Pharmacokinetic Study of Dispensing Granules and Standard Decoction of Cinnamomum cassia Twigs. Biomed. Chromatogr. 33, e4534. 10.1002/bmc.4534 30874318

[B196] Thongson C. Davidson P. M. Mahakarnchanakul W. Weiss J. (2004). Antimicrobial Activity of Ultrasound-Assisted Solvent-Extracted Spices. Lett. Appl. Microbiol. 39, 401–406. 10.1111/j.1472-765x.2004.01605.x 15482429

[B197] Tsai I.-L. Hung C.-H. Duh C.-Y. Chen I.-S. (2002). Cytotoxic Butanolides and Secobutanolides from the Stem Wood of Formosan Lindera Communis. Planta Med. 68, 142–145. 10.1055/s-2002-20260 11859465

[B198] Tsatsakis A. Docea A. O. Calina D. Tsarouhas K. Zamfira L.-M. Mitrut R. (2019). A Mechanistic and Pathophysiological Approach for Stroke Associated with Drugs of Abuse. Jcm 8, 1295. 10.3390/jcm8091295 PMC6780697 31450861

[B199] Tsuji-Naito K. (2008). Aldehydic Components of Cinnamon Bark Extract Suppresses RANKL-Induced Osteoclastogenesis through NFATc1 Downregulation. Bioorg. Med. Chem. 16, 9176–9183. 10.1016/j.bmc.2008.09.036 18823786

[B200] Tung Y.-T. Yen P.-L. Lin C.-Y. Chang S.-T. (2010). Anti-inflammatory Activities of Essential Oils and Their Constituents from Different Provenances of Indigenous Cinnamon (Cinnamomum Osmophloeum) Leaves. Pharm. Biol. 48, 1130–1136. 10.3109/13880200903527728 20815702

[B201] Turgis M. Vu K. D. Dupont C. Lacroix M. (2012). Combined Antimicrobial Effect of Essential Oils and Bacteriocins against Foodborne Pathogens and Food Spoilage Bacteria. Food Res. Int. 48, 696–702. 10.1016/j.foodres.2012.06.016

[B202] Ultee A. Kets E. P. W. Smid E. J. (1999). Mechanisms of Action of Carvacrol on the Food-Borne Pathogen *Bacillus* Cereus. Appl. Environ. Microbiol. 65, 4606–4610. 10.1128/aem.65.10.4606-4610.1999 10508096 PMC91614

[B203] Ungureanu A. Zlatian O. Mitroi G. Drocaş A. Ţîrcă T. Călina D. (2017). Staphylococcus aureus Colonisation in Patients from a Primary Regional Hospital. Mol. Med. Rep. 16, 8771–8780. 10.3892/mmr.2017.7746 29039613 PMC5779955

[B204] Unlu M. Ergene E. Unlu G. V. Zeytinoglu H. S. Vural N. (2010). Composition, Antimicrobial Activity and In Vitro Cytotoxicity of Essential Oil from Cinnamomum Zeylanicum Blume (Lauraceae). Food Chem. Toxicol. 48, 3274–3280. 10.1016/j.fct.2010.09.001 20828600

[B205] Utchariyakiat I. Surassmo S. Jaturanpinyo M. Khuntayaporn P. Chomnawang M. T. (2016). Efficacy of Cinnamon Bark Oil and Cinnamaldehyde on Anti-multidrug Resistant Pseudomonas aeruginosa and the Synergistic Effects in Combination with Other Antimicrobial Agents. BMC Complement. Altern. Med. 16, 158. 10.1186/s12906-016-1134-9 27245046 PMC4888607

[B206] Vafa M. Mohammadi F. Shidfar F. Sormaghi M. S. Heidari I. Golestan B. (2012). Effects of Cinnamon Consumption on Glycemic Status, Lipid Profile and Body Composition in Type 2 Diabetic Patients. Int. J. Prev. Med. 3, 531–536. 22973482 PMC3429799

[B207] Vallianou N. Tsang C. Taghizadeh M. Davoodvandi A. Jafarnejad S. (2019). Effect of Cinnamon (Cinnamomum Zeylanicum) Supplementation on Serum C-Reactive Protein Concentrations: A Meta-Analysis and Systematic Review. Complement. therapies Med. 42, 271–278. 10.1016/j.ctim.2018.12.005 30670254

[B208] Vallverdú-Queralt A. Regueiro J. Martínez-Huélamo M. Rinaldi Alvarenga J. F. Leal L. N. Lamuela-Raventos R. M. (2014). A Comprehensive Study on the Phenolic Profile of Widely Used Culinary Herbs and Spices: Rosemary, Thyme, Oregano, Cinnamon, Cumin and Bay. Food Chem. 154, 299–307. 10.1016/j.foodchem.2013.12.106 24518346

[B209] Vanschoonbeek K. Thomassen B. J. W. Senden J. M. Wodzig W. K. W. H. Van Loon L. J. C. (2006). Cinnamon Supplementation Does Not Improve Glycemic Control in Postmenopausal Type 2 Diabetes Patients. J. Nutr. 136, 977–980. 10.1093/jn/136.4.977 16549460

[B210] Verspohl E. J. Bauer K. Neddermann E. (2005). Antidiabetic Effect ofCinnamomum cassia andCinnamomum Zeylanicum *In vivo* andIn Vitro. Phytother. Res. 19, 203–206. 10.1002/ptr.1643 15934022

[B211] Vivas A. P. M. Migliari D. A. (2015). Cinnamon-induced Oral Mucosal Contact Reaction. Todentj 9, 257–259. 10.2174/1874210601509010257 PMC4541332 26312097

[B212] Walanj S. Walanj A. Mohan V. Thakurdesai P. A. (2014). Efficacy and Safety of the Topical Use of Intranasal Cinnamon Bark Extract in Seasonal Allergic Rhinitis Patients: A Double-Blind Placebo-Controlled Pilot Study. J. Herbal Med. 4, 37–47. 10.1016/j.hermed.2013.12.002

[B213] Wang H.-M. Chen C.-Y. Wen Z.-H. (2011). Identifying Melanogenesis Inhibitors from Cinnamomum Subavenium with In Vitro and In Vivo Screening Systems by Targeting the Human Tyrosinase. Exp. Dermatol. 20, 242–248. 10.1111/j.1600-0625.2010.01161.x 21054558

[B214] Wang J. Su B. Jiang H. Cui N. Yu Z. Yang Y. (2020). Traditional Uses, Phytochemistry and Pharmacological Activities of the Genus Cinnamomum (Lauraceae): A Review. Fitoterapia 146, 104675. 10.1016/j.fitote.2020.104675 32561421

[B215] Wang R. Wang R. Yang B. (2009). Extraction of Essential Oils from Five Cinnamon Leaves and Identification of Their Volatile Compound Compositions. Innovative Food Sci. Emerging Tech. 10, 289–292. 10.1016/j.ifset.2008.12.002

[B216] Wannissorn B. Jarikasem S. Siriwangchai T. Thubthimthed S. (2005). Antibacterial Properties of Essential Oils from Thai Medicinal Plants. Fitoterapia 76, 233–236. 10.1016/j.fitote.2004.12.009 15752638

[B217] Wansi S. L. Nyadjeu P. Ngamga D. Mbuyo E. P. N. Nguelefack T. B. Kamanyi A. (2007). Blood Pressure Lowering Effect of the Ethanol Extract from the Stembark of Cinnamomum Zeylanicum (Lauraceae) in Rats. Pharmacol. Online 3, 166–176.

[B218] Wild S. Roglic G. Green A. Sicree R. King H. (2004). Global Prevalence of Diabetes: Estimates for the Year 2000 and Projections for 2030. Diabetes Care 27, 1047–1053. 10.2337/diacare.27.5.1047 15111519

[B219] Wiweko B. Susanto C. A. (2017). The Effect of Metformin and Cinnamon on Serum Anti-mullerian Hormone in Women Having PCOS: A Double-Blind, Randomized, Controlled Trial. J. Hum. Reprod. Sci. 10, 31–36. 10.4103/jhrs.JHRS_90_16 28479753 PMC5405645

[B220] Woehrlin F. Fry H. Abraham K. Preiss-Weigert A. (2010). Quantification of Flavoring Constituents in Cinnamon: High Variation of Coumarin in cassia Bark from the German Retail Market and in Authentic Samples from Indonesia. J. Agric. Food Chem. 58, 10568–10575. 10.1021/jf102112p 20853872

[B221] Wondrak G. Villeneuve N. F. Lamore S. D. Bause A. S. Jiang T. Zhang D. D. (2010). The Cinnamon-Derived Dietary Factor Cinnamic Aldehyde Activates the Nrf2-dependent Antioxidant Response in Human Epithelial Colon Cells. Molecules 15, 3338–3355. 10.3390/molecules15053338 20657484 PMC3101712

[B222] Wu K. Lin Y. Chai X. Duan X. Zhao X. Chun C. (2019). Mechanisms of Vapor-phase Antibacterial Action of Essential Oil from Cinnamomum Camphora Var. Linaloofera Fujita against *Escherichia coli* . Food Sci. Nutr. 7, 2546–2555. 10.1002/fsn3.1104 31428342 PMC6694428

[B223] Wu V. Qiu X. Delosreyes B. Lin C. Pan Y. (2009). Application of Cranberry Concentrate (Vaccinium Macrocarpon) to Control *Escherichia coli* O157:H7 in Ground Beef and its Antimicrobial Mechanism Related to the Downregulated Slp, hdeA and Cfa. Food Microbiol. 26, 32–38. 10.1016/j.fm.2008.07.014 19028302

[B224] Yanakiev S. (2020). Effects of Cinnamon (Cinnamomum spp.) in Dentistry: A Review. Molecules 25. 10.3390/molecules25184184 PMC7571082 32932678

[B225] Yang C.-H. Li R.-X. Chuang L.-Y. (2012). Antioxidant Activity of Various Parts of Cinnamomum cassia Extracted with Different Extraction Methods. Molecules 17, 7294–7304. 10.3390/molecules17067294 22695234 PMC6268419

[B226] Yang S. Y. Wang H. M. Wu T. W. Chen Y. J. Shieh J. J. Lin J. H. (2013). Subamolide B Isolated from Medicinal Plant Cinnamomum Subavenium Induces Cytotoxicity in Human Cutaneous Squamous Cell Carcinoma Cells through Mitochondrial and CHOP-dependent Cell Death Pathways. Evid. Based Complement. Alternat Med. 2013, 630415. 10.1155/2013/630415 23573140 PMC3610371

[B227] Yang Y.-C. Lee H.-S. Lee S. H. Clark J. M. Ahn Y.-J. (2005). Ovicidal and Adulticidal Activities of Cinnamomum Zeylanicum Bark Essential Oil Compounds and Related Compounds against Pediculus Humanus Capitis (Anoplura: Pediculicidae). Int. J. Parasitol. 35, 1595–1600. 10.1016/j.ijpara.2005.08.005 16188263

[B228] Yap P. S. X. Krishnan T. Chan K.-G. Lim S. H. E. (2015). Antibacterial Mode of Action of Cinnamomum Verum Bark Essential Oil, Alone and in Combination with Piperacillin, against a Multi-Drug-Resistant *Escherichia coli* Strain. J. Microbiol. Biotechnol. 25, 1299–1306. 10.4014/jmb.1407.07054 25381741

[B229] Yeh R.-Y. Shiu Y.-L. Shei S.-C. Cheng S.-C. Huang S.-Y. Lin J.-C. (2009). Evaluation of the Antibacterial Activity of Leaf and Twig Extracts of Stout Camphor Tree, Cinnamomum Kanehirae, and the Effects on Immunity and Disease Resistance of White Shrimp, *Litopenaeus* Vannamei. Fish Shellfish Immunol. 27, 26–32. 10.1016/j.fsi.2008.11.008 19063975

[B230] Yun J.-W. You J.-R. Kim Y.-S. Kim S.-H. Cho E.-Y. Yoon J.-H. (2018). *In vitro* and In Vivo Safety Studies of Cinnamon Extract ( Cinnamomum cassia ) on General and Genetic Toxicology. Regul. Toxicol. Pharmacol. 95, 115–123. 10.1016/j.yrtph.2018.02.017 29501463

[B231] Zhang J.-H. Liu L.-Q. He Y.-L. Kong W.-J. Huang S.-A. (2010). Cytotoxic Effect of Trans-cinnamaldehyde on Human Leukemia K562 Cells. Acta Pharmacol. Sin 31, 861–866. 10.1038/aps.2010.76 20581850 PMC4007726

[B232] Zhang W. Xu Y.-c. Guo F.-j. Meng Y. Li M.-l. (2008). Anti-diabetic Effects of Cinnamaldehyde and Berberine and Their Impacts on Retinol-Binding Protein 4 Expression in Rats with Type 2 Diabetes Mellitus. Chin. Med. J. 121, 2124–2128. 10.1097/00029330-200811010-00003 19080170

[B233] Zhao J. Ma J.-S. (2016). Phytochemicals and Biological Activities of Genus Cinnamomum. Res. Rev. J. Pharmacognosy Phytochemistry 4, 27–34.

[B234] Zimmet P. Alberti K. G. M. M. Shaw J. (2001). Global and Societal Implications of the Diabetes Epidemic. Nature 414, 782–787. 10.1038/414782a 11742409

[B235] Zlatian O. Balasoiu A. T. Balasoiu M. Cristea O. Docea A. O. Mitrut R. (2018). Antimicrobial Resistance in Bacterial Pathogens Among Hospitalised Patients with Severe Invasive Infections. Exp. Ther. Med. 16, 4499–4510. 10.3892/etm.2018.6737 30542398 PMC6257814

